# cAMP–PKA/EPAC signaling pathways: crucial regulators of lipid homeostasis

**DOI:** 10.1080/21623945.2025.2603605

**Published:** 2026-01-07

**Authors:** Caixia Chen, Hui Gao, Qi Tian, Junwei Cao

**Affiliations:** a College of Life Sciences, Inner Mongolia Agricultural University, Inner Mongolia Key Laboratory of Biomanufacturing Techenology, Inner Mongolia Autonomous Region Endemic Livestock Biotechnology Innovation Team, Hohhot, China; b Clinical Medicine Research Center, Affiliated Hospital of Inner Mongolia Medical University, Inner Mongolia Autonomous Region Key Laboratory of Cell Biology, Hohhot, China; c Department of Thoracic Surgery, Inner Mongolia Hospital, Peking University Cancer Hospital, Hohhot, China

**Keywords:** Cyclic adenosine monophosphate (cAMP), protein kinase a (PKA), exchange proteins directly activated by cAMP (EPAC), homeostasisoins homoeostasis-related diseases, adipose microenvironment

## Abstract

Adipose omeostasishomoeostasis is maintained through the precise coordination of lipogenesis, lipolysis, and adipocyte differentiation, with microenvironmental components dynamically regulating lipid metabolism. Even though the classical cAMP-PKA pathway has been well-characterized for its function in lipid metabolism by phosphorylating transcription factors and lipolytic enzymes, little is known about how it collaborates with elements of the adipose tissue microenvironment, such as immune cells and the vascular endothelium, especially in pathological situations like obesity. EPAC, a newly discovered cAMP effector, has shown new signalingsignallingsignalling signalling pathways in the immune and cardiovascular systems by activating small G proteins. However, there are important understanding gaps regarding its roles in adipose metabolism, namely adipocyte development, microenvironmental interaction, and the pathophysiology of metabolic diseases. By bringing together disparate studies on PKA and EPAC, this review provides the first comprehensive synthesis of the cAMP-PKA/EPAC dual signaling signalling signallingcins signalling network, filling in knowledge gaps. The reciprocal regulation between this signaling signalling signalling signalling network and the adipose microenvironment establishes a novel ‘signaling-microenvironment-systemic metabolism’ framework for understanding metabolic disorders, including obesity, diabetes, and hepatic steatosis. Pharmacological modulation of the PKA/EPAC signalingsignalling signalling signalling pathways may therefore represent a viable therapeutic approach for restoring adipose tissue homeostasis homoeostasis.

## 1. Introduction

cAMP is an important second messenger within cells that is generated from adenosine triphosphate (ATP) by the action of adenylate cyclase (AC). Its molecular structure consists of an adenine, a ribose, and a phosphate group. The phosphate group forms a cyclic structure with the 3′ and 5′ carbon atoms of the ribose. This unique structure enables cAMP to specifically interact with various intracellular target molecules, participating in processes such as cell proliferation, apoptosis, differentiation, hormone secretion, cytoskeleton organization, and metabolism [1–6]. The main downstream targets of cAMP include cAMP-dependent PKA, EPAC, and ion channels. cAMP participates in various pathological processes through its downstream targets, including tumours, neurological diseases, cardiovascular diseases, and inflammation [7–10]. As a well-known intracellular second messenger, cAMP regulates the activity of a series of metabolic enzymes, transcription factors, and regulatory factors by activating PKA or EPAC, thereby affecting lipid deposition and metabolism [11–13]. For example, the cAMP – PKA/EPAC pathway influences lipid homoeostasis by regulating acetyl-CoA carboxylase (ACC), fatty acid synthase, cAMP-responsive element-binding protein (CREB), peroxisome proliferator-activated receptor (PPAR)α, and AMP-activated protein kinase. Additionally, cAMP signalling not only participates in regulating the energy metabolism balance of adipocytes but also affects the mitochondrial function and activity of adipocytes, maintaining the body’s energy homoeostasis [14–18]. Therefore, cAMP plays a crucial role in maintaining the normal function of adipocytes and the balance of lipid metabolism. Once cAMP signalling becomes abnormal, adipose homoeostasis is disrupted, leading to a series of metabolic disorders, including obesity and diabetes [19,20]. Additionally, in the microenvironment of adipose tissue, cAMP signalling is finely regulated. Adipocytes interact with surrounding preadipocytes, immune cells, fibroblasts, endothelial cells, and other cells, forming a complex intercellular communication network. For example, immune cells, particularly macrophages, can secrete various cytokines, including macrophage migration inhibitory factor. Migration inhibitory factors affects lipolytic enzymes and adipocyte hypertrophy [21]. And the absence of migration inhibitory factors exacerbates the impact of a high-energy fructose diet on lipid accumulation in the mouse liver [22]. Adipose tissue can secrete various cytokines and chemokines, such as leptin, adiponectin, and resistin. Additionally, adipose tissue secretes chemokines, such as monocyte chemoattractant protein-1, which attract immune cells to infiltrate the adipose tissue [23]. Various signalling molecules are present in adipose tissue, such as insulin, insulin-like growth factor-1, and adrenergic signals, which regulate the metabolism, proliferation, and differentiation of adipocytes, as well as the function of immune cells, by binding to their respective receptors [24]. Moreover, the regulation of fat homoeostasis by cAMP is not static but involves a complex dynamic balance. The direction and degree of this regulation are influenced by various factors, including nutrition, hormone levels, and the different physiological and pathological states of adipose tissue. For example, in a fasting state, the level of glucagon in the body increases, which binds to receptors on the surface of adipocytes, activating AC, increasing intracellular cAMP levels, and promoting lipolysis to provide energy for the body. After eating, insulin secretion increases, inhibiting AC activity, lowering cAMP levels, and promoting fat synthesis and storage. Additionally, phosphodiesterase (PDE) inhibitors can increase intracellular cAMP levels by inhibiting cAMP hydrolysis, potentially regulating fat metabolism and improving obesity and diabetes-related metabolic disorders in animal experiments and some preclinical studies [25,26]. Owing to the important regulatory role of cAMP signalling in fat homoeostasis, intervention strategies targeting the cAMP – PKA/EPAC signalling pathway have significant potential therapeutic value. This article reviews the latest research progress on cAMP – PKA/EPAC in adipose homoeostasis and discusses the complex functions of cAMP signalling in the adipose microenvironment. Considering the importance of the cAMP – PKA/EPAC signalling pathway in adipose biology, selectively targeting PKA, EPAC, PDEs, or ACs – and modulating cAMP levels in the adipose microenvironment – represents a promising therapeutic strategy. Regulating the cAMP signalling pathway to improve adipose homoeostasis is a highly promising research direction for the prevention and treatment of metabolic diseases such as obesity, diabetes, and fatty liver.

## 2. cAMP signalling pathway

cAMP is deeply involved in numerous physiological and pathological processes [27,28]. cAMP originates from two pathways: transmembrane adenylyl cyclases (tmACs, Adcy1–9) in the plasma membrane (regulated by G proteins, hormones, and neurotransmitters) and soluble adenylyl cyclases (sACs, Adcy10) in the cytoplasm/organelles (G protein-insensitive) [29–31]. PDEs are crucial for cAMP signal transduction [32]. Members of the PDE family have similar domains, typically including a catalytic domain responsible for hydrolysing phosphodiester bonds, as well as multiple regulatory domains for substrate binding, enzyme activity regulation, and interactions with other proteins. Different types of PDEs exhibit certain differences in amino acid sequences and structures, which determine their substrate specificity, regulatory characteristics, and tissue distribution. The main function of PDEs is to degrade cAMP and cGMP, thereby terminating or modulating the cell signalling pathways mediated by these second messengers. By hydrolysing cAMP and cGMP, PDEs can regulate the intracellular concentrations of these signalling molecules, thereby affecting various physiological functions of the cells, such as cell proliferation, differentiation, apoptosis, metabolism, ion channel activity, and gene expression [33–37]. PDEs can be divided into 11 different families (PDE1–PDE11) on the basis of their substrate specificity, amino acid sequence homology, enzymatic kinetic properties, and regulatory mechanisms. Among these, three selectively hydrolyse cAMP (PDE4, 7, and 8), whereas five hydrolyse both cAMP and cGMP (PDE1, 2, 3, 10, and 11) [38]. Currently, four effectors of cAMP have been identified in eukaryotes: PKA, EPAC, cyclic nucleotide-gated channels, and proteins containing the Popeye domain [39]. PKA and EPAC are the two most important effectors through which cAMP exerts its functions. The cAMP pathway plays a central role in adipose tissue differentiation and metabolism: it accelerates adipocyte differentiation via PKA/EPAC-dependent pathways [40,41]. For example, cAMP drives the browning of adipocytes through a biphasic mechanism [42]. First, cAMP activates inflammatory signals, cytoskeletal reorganization, and angiogenesis-related pathways, laying the foundation for metabolic remodelling. Subsequently, it fully activates the thermogenic program through chromatin accessibility remodelling and transcriptome reprogramming, inducing the transformation of white adipose tissue (WAT) into beige/brown adipocytes with thermogenic functions. This process relies on the coordinated regulation of the transcription factor nuclear factor interleukin 3 [43]. Lipolysis regulation involves Gs-coupled GPCRs activating AC to produce cAMP from ATP. Once the intracellular cAMP level increases, it promotes the phosphorylation of PKA. Activated PKA subsequently induces the phosphorylation of hormone-sensitive lipase (HSL) and perilipin 1 while also activating adipose triglyceride lipase (ATGL), ultimately initiating the lipolysis process [44,45]. Strict submicron-scale control of cAMP concentration ensures lipolysis specificity and intracellular fat homoeostasis [46]. As an upstream regulatory hub, GPCRs regulate cAMP production by activating/inhibiting heterotrimeric G proteins, thereby controlling the dynamic balance between lipogenesis (such as the esterification pathway) and lipolysis. For example, norepinephrine released by the sympathetic nervous system activates the cAMP signalling pathway through β-adrenergic receptors, simultaneously promoting lipolysis and browning, whereas insulin inhibits lipolysis and promotes fat storage by suppressing cAMP production. This regulatory network not only determines the energy state of adipocytes but also influences systemic metabolic sensitivity through the secretion of adipokines like adiponectin and leptin, forming a microenvironmental ‘fat-organ’ crosstalk network [47]. In energy allocation, cAMP-driven browning dissipates WAT energy as heat to resist diet-induced obesity; activated brown adipose tissue (BAT) uses uncoupling protein 1 (UCP1) to transport fatty acids into mitochondria for heat production, forming a ‘lipolysis – thermogenesis’ axis [48]. Dietary patterns also regulate cAMP signalling: a high-protein diet enhances cAMP signalling by increasing the glucagon/insulin ratio, inhibiting fat accumulation, whereas a carbohydrate-rich diet strengthens the insulin pathway, promoting fat synthesis and storage [49]. In the microenvironment of adipose tissue, excessive lipid load in adipocytes can trigger endoplasmic reticulum stress, recruit immune cells, and induce chronic inflammation, whereas cAMP alleviates intracellular lipid accumulation and mitigates the inflammatory cascade by promoting lipolysis and browning [50]. Therefore, the cAMP pathway integrates metabolic signals (such as dietary sensing and hormonal regulation) with microenvironmental homoeostasis, becoming a core node in maintaining the plasticity of adipose tissue function. Its mechanism provides key targets for intervention in obesity and metabolic diseases.

## 3. PKA

**Figure 1.**
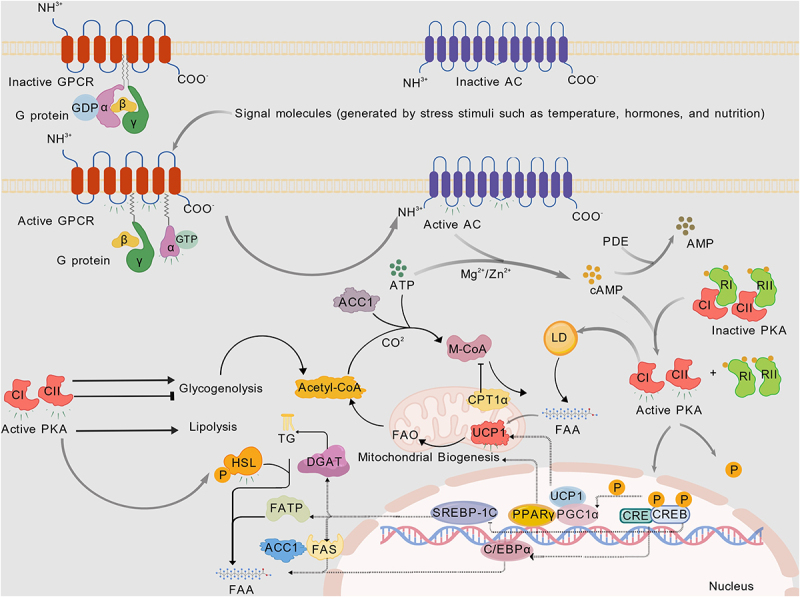
Diagram of the cAMP – PKA pathway involved in lipid homoeostasis created with BioGDP.com. Extracellular stimuli (temperature, hormones, nutrients) activate GPCRs, triggering G protein/AC signalling to produce cAMP. cAMP activates PKA, which phosphorylates downstream targets to regulate glycogenolysis and lipolysis. Metabolites feed into cellular metabolism; nuclear transcription factors (e.g. UCP1 regulators) drive mitochondrial biogenesis/thermogenesis. CPT1α mediates mitochondrial fatty acid oxidation, coordinating glucose/lipid metabolism and thermogenesis to sustain lipid homoeostasis. Gray arrows denote pathway activation/promotion. Gray arrows:Pathway activation/promotion dashed lines:Transcriptional regulation/protein expression black solid lines:Metabolic pathways →:Positive regulation/activation ⊣:Negative regulation/inhibition lightning bolts:Activated molecular states.

PKA is a heterotetramer composed of two regulatory subunits (RI/RII-α or RI/RII-β) and two catalytic subunits (isoforms-Cα, Cβ, Cγ, PRKX) [51]. The R subunit is mainly used to bind cAMP while inhibiting the activity of the C subunit. The C subunit has kinase activity and can phosphorylate the serine/threonine residues of substrate proteins. In the absence of cAMP, the R subunit binds to the C subunit, inhibiting kinase activity. When cAMP binds to the R subunit, it causes a conformational change in the R subunit, releasing the C subunit, which then phosphorylates downstream target proteins. By phosphorylating downstream target proteins, it regulates various cellular functions, including metabolism, gene expression, cell proliferation, and differentiation [52–54]. The cAMP – PKA pathway plays a role in regulating lipid metabolism in multiple organs. First, the cAMP – PKA signalling pathway plays an important role in hepatic lipid homoeostasis [55]. Elevated cAMP levels inhibit lipogenesis through PKA-dependent inhibitory phosphorylation of fatty acid biosynthesis enzymes – ACC1 and pyruvate dehydrogenase [55,56]. A study indicates that inhibiting ACC1 activity can alleviate hepatic steatosis [57]. CREB is an important downstream effector of cAMP/PKA. PKA phosphorylates the Ser133 site of CREB, which then translocates from the cytoplasm to the nucleus, binds to CRE, and recruits coactivators (such as CBP/p300) to initiate transcription [58]. PKA-activated CREB inhibits the expression of genes that promote lipid synthesis in the liver, such as PPARγ and sterol regulatory element-binding protein-1c (SREBP-1C) [59,60]. This finding is consistent with previous findings that the activation of the cAMP/CREB pathway helps reduce hepatic lipid accumulation. LncRNA GAS5 knockdown mitigates hepatic lipid accumulation by regulating miR-26a-5p/PDE4B to activate the cAMP/CREB pathway. Furthermore, the activation of PPARγ coactivator-1α (PGC-1α) mediated by CREB, as well as the CREB-dependent inhibition of PPARγ, can enhance mitochondrial fatty acid oxidation [61]. A similar study showed that cAMP activation of PKA inhibits lipogenesis and promotes fatty acid oxidation [62]. In adipose tissue, cAMP activates PKA, promoting the translocation of HSL and the removal of the protective protein perilipin-1 from the surface of lipid droplets. This process can lead to an increase in lipolysis [63]. In animal experiments, activated PKA can phosphorylate CREB, regulating cell metabolism, proliferation, and differentiation, thereby promoting lipolysis, reducing lipogenesis, and ultimately helping to reduce fat deposition in fattening pigs [64]. In drug development, the natural compounds sudachitin and nobiletin stimulate lipolysis by activating the cAMP/PKA/HSL pathway in 3T3-L1 adipocytes, promoting the phosphorylation and activation of HSL and significantly enhancing lipolytic capacity [44]. The traditional Chinese medicine Fuzi improves lipid metabolism disorders in mice with Yang deficiency and hyperlipidaemia by modulating the PKA – PPARα–carnitine palmitoyltransferase 1α (CPT1α) pathway. Its mechanism is closely related to the activation of PPARα and promotion of CPT1α-mediated fatty acid mitochondrial oxidation [65]. These findings indicate that the lipolytic action of the cAMP – PKA signalling pathway in adipose tissue has certain clinical translational potential. However, a study has reported that CREB1 can promote the synthesis of monounsaturated fatty acids and the accumulation of triacylglycerol in goat mammary epithelial cells [66]. Moreover, the activation of the PKA pathway can increase the expression of PPARγ and lipid synthesis in differentiated human meibomian gland epithelial cells [67]. However, the regulation of lipid metabolism by cAMP-PKA can be context-dependent. For example, CREB1 promotes monounsaturated fatty acid synthesis and triacylglycerol accumulation in goat mammary epithelial cells [66], and PKA activation increases PPARγ expression and lipid synthesis in differentiated human meibomian gland epithelial cells [67]. These differential effects may be attributed to tissue specificity and other factors. Additionally, endoplasmic reticulum stress stimuli can induce lipolysis in adipocytes through the synergistic activation of the cAMP – PKA and extracellular signal-regulated kinase 1/2 (ERK1/2) signalling pathways [68]. These findings suggest that cAMP – PKA may interfere with other signalling pathways, jointly regulating lipid homoeostasis.Energy balance is crucial for maintaining lipid homoeostasis [69]. Mitochondria, as the ‘energy factories’ of the cell, directly determine the efficiency of energy metabolism through their quantity and function, while the state of energy metabolism, in turn, regulates the generation of mitochondria by signalling pathways. This dynamic balance is the core mechanism by which cells adapt to environmental changes and maintain homoeostasis. WAT browning is an adaptive mechanism that enables the body to cope with an energy surplus, converting ‘storage fat’ into ‘energy-consuming fat’ by activating thermogenic programs. The cAMP – PKA signalling pathway can promote the browning of white fat/activation of brown fat by regulating the expression of downstream CREB. In vitro study has shown that increased PKA expression leads to an increase in mitochondrial content, particularly enhancing the energy metabolism of adipocytes [70]. Additionally, PKA activates the mRNA and protein expression of its downstream target CREB and increases the phosphorylation level of CREB, promoting the browning of white fat and enhancing mitochondrial biogenesis [71]. In muscles, lactate injection inhibits skeletal muscle lipolysis while promoting triglyceride accumulation and lipogenesis, a process regulated by the cAMP – PKA pathway [72]. Additionally, lactate injection increased the expression and activity of citrate synthase, suggesting an increase in the mitochondrial content [73]. cAMP-regulated transcription coactivator 3 (CRTC3) is a coactivator of CREB and can mediate the function of the PKA signalling pathway. The overexpression of CRTC3 can promote the adipogenic differentiation of intramuscular and subcutaneous adipocytes in pigs, which participate in various biological processes, including lipid and energy homoeostasis [74]. CRTC3 promotes lipid accumulation and increases the expression of PPARγ, C/EBPα, leptin, and fatty acid binding protein 4 (FABP4) while decreasing the expression of ATGL and HSL in intramuscular adipocytes [75]. Similarly, another study showed that CRTC3 regulates lipid metabolism and adipocyte differentiation in intramuscular and subcutaneous adipocytes by activating the calcium pathway [76]. CREBH is encoded by CREB3L3, which has high homology with the CREB/activating transcription factor molecular family through its b-Zip domain [77]. It is expressed primarily in the liver, adipose tissue, and intestinal epithelial cells and is closely related to metabolic regulation. A study found that CREBH plays an important role in lipid homoeostasis [78]. The phosphorylation activation of CREBH at the serine-133 site transcriptionally activates genes involved in triglyceride metabolism, thereby inhibiting plasma triglyceride metabolism. Its functions include the oxidation of fatty acids, phagocytosis of fats, and expression of apolipoproteins related to lipoprotein lipase activation and fat generation inhibition [79]. CREBH and the apolipoprotein AIV are involved in the production of larger lipoproteins [80]. CREBH plays a key role in metabolic regulation, particularly in lipid metabolism. Its phosphorylation, activation and interaction with apolipoproteins are highly important for maintaining normal metabolic homoeostasis.In summary, cAMP regulates lipogenesis, lipolysis, and energy metabolism via PKA and downstream molecules (ACC1, HSL, PPARα, CREB, CRTC3, CREBH), with tissue-specific bidirectional regulatory effects (Figure 1) [81].

## 4. EPAC

**Figure 2.**
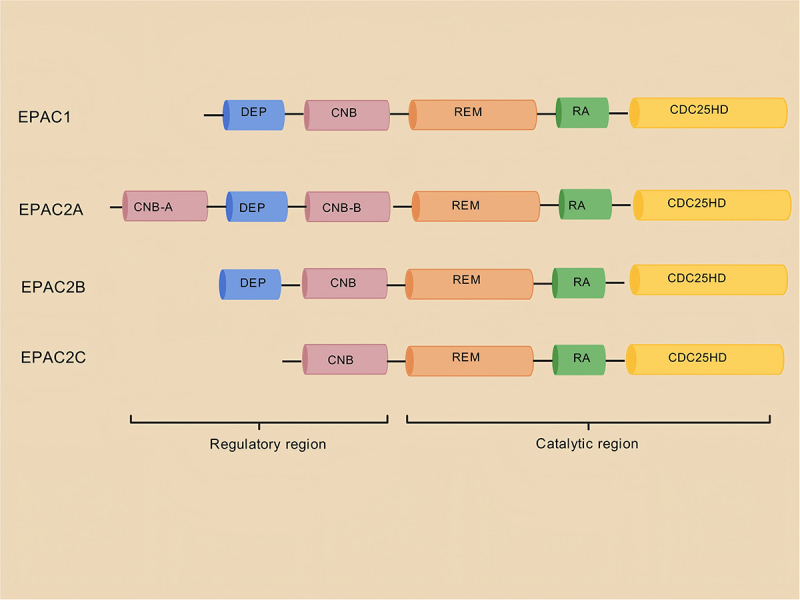
Schematic of the structure and composition of EPAC isoforms created with BioGDP.com. CNB: cyclic nucleotide-binding domain responsible for binding cAMP. dep: dishevelled, EGL-10, and pleckstrin homology domains involved in protein – protein interactions or subcellular localization. REM: a Ras exchange motif that mediates interactions with small G proteins (such as Rap1). RA: Ras association domain, assisting in protein – protein interactions. CDC25HD: the CDC25 homology domain is a catalytic domain of EPAC that activates downstream small G proteins.

Unlike PKA, which is oriented towards catabolic processes, EPAC-mediated cAMP signalling is more involved in the regulation of adipogenesis, cell differentiation and secretory functions. This functional divergence stems from its unique molecular mechanism as a Rap guanine nucleotide exchange factor (RapGEF). The EPAC protein family currently includes EPAC1, EPAC2, and REPAC [30,82,83]. EPAC1 and EPAC2 function as GEFs for the small GTPases Rap1 and Rap2, operating independently of PKA. When bound to cAMP, EPAC promotes the exchange of guanosine diphosphate (GDP) with guanosine triphosphate (GTP), thereby inducing the activation of the small G protein Rap [30,83]. In contrast, the Rap – GTPase-activating protein enhances the intrinsic GTP hydrolysis activity of Rap, leading to GTPase inactivation. The cycling of Rap between its inactive and active states provides a mechanism for regulating its binding with effector proteins. REPAC, lacking the regulatory sequences of EPAC1/2, has a unique regulation and can activate both Rap1A and Ras, though research on REPAC remains limited [84]. EPAC1, EPAC2, and REPAC are encoded by the RAPGEF3, RAPGEF4, and RAPGEF5 genes, respectively. EPAC1 and EPAC2 are cAMP-dependent RapGEFs. EPAC2 (RAPGEF4) has three main splice variants: EPAC2A, EPAC2B, and EPAC2C [85]. Their structural differences are reflected mainly in the N-terminal regulatory region, leading to distinct subcellular localizations and functional regulatory characteristics (Figure 2). Structurally, EPAC1 and EPAC2 contain an autoinhibitory N-terminal regulatory region and a C-terminal catalytic region. In the N-terminal regulatory region of the domain, EPAC1 contains one cAMP-binding domain (CBD), whereas EPAC2 contains two (CBD-A and CBD-B). The DEP domain mainly mediates membrane localization (such as interactions with phospholipids), and EPAC2 additionally has an N-terminal autoinhibitory sequence. The central hinge region, also known as the helical linker region, primarily transmits cAMP-induced conformational changes, relieving autoinhibition. The C-terminal catalytic domain contains a RapGEF (CDC25 homolog) domain, which directly binds to Rap1/2 and catalyzes GDP release, and a conserved ‘GEF catalytic loop’. The RA domain (only in EPAC2) provides an additional interaction interface with small G proteins such as Ras.

**Figure 3.**
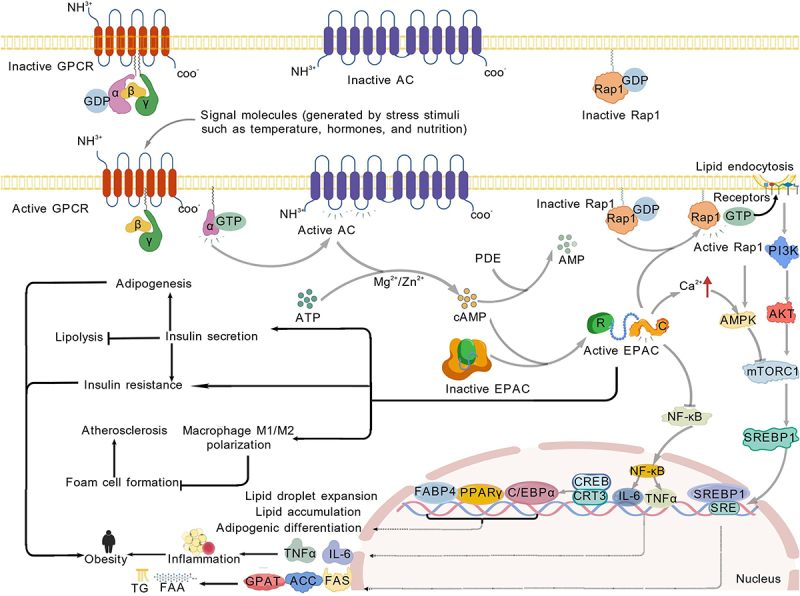
Diagram of the cAMP–EPAC pathway involved in lipid homoeostasis created with BioGDP.com. Extracellular stimuli (temperature, hormones, nutrition) activate GPCRs, driving G protein/AC signalling to generate cAMP. cAMP activates EPAC, which promotes Rap1 activation; this cascade regulates lipid uptake (via PI3K/AMPK/mTORC1 pathways) and modulates cellular metabolism. Dysregulated lipid processes (lipid droplet expansion, fat accumulation) and M1/M2 macrophage polarization imbalance trigger inflammatory factor (e.g. TNF-α, IL-6) release, activating NF-κB/SREBP. This forms a ‘metabolism – inflammation’ vicious cycle, contributing to obesity, atherosclerosis, and foam cell formation – illustrating the cAMP–EPAC pathway’s role in maintaining lipid homoeostasis.

EPAC promotes adipocyte differentiation by increasing the expression of key adipogenic transcription factors (PPARγ, C/EBPα) and downstream targets (e.g. FABP4), regulating lipid droplet formation and preadipocyte maturation. For example, CPNX14 (a synthetic analog of phoenixin-14) has been shown to stimulate lipogenesis through an EPAC-dependent mechanism, and EPAC-specific inhibitors can completely block its lipogenic effect [86]. EPAC activates the ERK signalling pathway, which is crucial for early lipogenic events, including the induction of PPARγ. This mechanism is consistent with the results of ERK inhibitors blocking the upregulation of PPARγ induced by CPNX14, further confirming the role of the EPAC – ERK signalling cascade in adipocyte differentiation. Similarly, phoenixin-14 (PNX-14) has also been reported to drive the differentiation of 3T3-L1 preadipocytes through a cAMP/EPAC-dependent mechanism, further supporting the importance of EPAC in fat accumulation and metabolic regulation [1]. This finding is consistent with previous reports showing that EPAC is involved in lipogenesis and lipolysis [87,88]. The activation of EPAC is of critical importance to obesity and its associated metabolic disorders [89]. The activated EPAC immediately initiates downstream cascades, activating numerous downstream effectors, which promote adipocyte hypertrophy and proliferation and drive adipose tissue expansion [90–92]. Lipid droplets, as important structural and functional units of adipocyte differentiation, not only serve as storage sites for triglycerides but also participate in metabolic regulation and signal transduction [93]. EPAC proteins may regulate lipid metabolism-related processes such as triglyceride synthesis, fatty acid uptake and intracellular transport, fatty acid synthesis and esterification, and the expression of lipid droplet-associated proteins by acting on downstream transcription factors such as PPARγ and SREBP1, thereby promoting lipid droplet expansion in breast cancer cells [94]. This finding is consistent with earlier findings that the action targets cAMP, PKA, and EPACs influence the expansion of lipid droplets. cAMP-mediated stimulation of adipocyte differentiation requires the synergistic action of EPAC- and cAMP-dependent protein kinase-dependent processes [95]. Moreover, cAMP stimulation can influence the exocytosis and secretion of adipokines in white adipocytes through the EPAC signalling pathway, independent of PKA [96]. Furthermore, EPAC interacts with other signalling molecules, such as promoting the internalization of P2×1 receptors or activating the reactivation of oxygen species-mediated lipid peroxidation and ferroptosis, thereby participating in lipid metabolism regulation [97,98]. These findings underscore the pivotal role of EPAC in adipocyte differentiation, maturation, and systemic lipid metabolism. EPAC has tissue-specific regulatory functions in maintaining lipid homoeostasis [16]. EPAC1 plays a crucial role in the transcriptional regulation of PGC-1α and related fatty acid metabolism, and EPAC deficiency affects lipid metabolism in skeletal muscle, leading to decreased exercise capacity [99]. In the heart, EPAC1 acts as a metabolic sensor, promoting lipotoxicity in cardiomyocytes [100]. In the pancreas, EPAC2A can increase insulin secretion, thereby affecting glucose and lipid metabolism [101]. EPAC2A plays a role in pancreatic β-cells, and EPAC2A in the hypothalamus may promote leptin signalling to regulate appetite and lipid homoeostasis in response to increased metabolic demands. A study found that the use of EPAC2A activators could be a new strategy for obesity management [102]. In contrast, a study revealed that EPAC2 is highly expressed in adipose tissue and promotes lipid accumulation [103]. Therefore, the bidirectional regulatory role of EPAC in lipid homoeostasis may be related to tissue specificity and EPAC subtypes. The activity of EPAC was found to be dependent on the lipid environment, such as palmitoylation, enhancing its membrane localization and function [100]. The lipid microenvironment also affects the function of its downstream effector molecules (such as Rab27) [104]. In summary, EPAC plays a bidirectional regulatory role in adipogenesis, lipid storage, metabolic homoeostasis, and diseases (such as obesity, breast cancer, and cardiovascular diseases) through a complex network involving multiple tissues and pathways (Figure 3). Its mechanisms include transcriptional regulation, signalling pathway crosstalk, and dynamic interactions with the lipid microenvironment.

Gray arrows:Pathway activation/promotion Dashed lines:Transcriptional regulation/protein expression Black solid lines:Metabolic pathways →:Positive regulation/activation ⊣:Negative regulation/inhibition Lightning bolts:Activated molecular states Red↑: Increase/elevation.

## 5. Role of cAMP – PKA/EPAC in lipid homoeostasis-related diseases

### 5.1. Obesity

According to epidemiological surveys and predictive study, by 2035, over half of the world’s population will be affected by overweight and obesity [105]. Obesity and related diseases (such as diabetes, cardiovascular diseases, and cancer) severely threaten human health and impose a significant economic burden [106–108]. A study indicates that cAMP levels are elevated in the plasma of obese patients [109]. However, another study has also shown that in the adipocytes of obese rats, the levels of cAMP is reduced. And the lipolytic function of WAT is impaired in the obese state, mainly due to the activation barrier of the cAMP signalling pathway [110]. So increasing cAMP levels in adipocytes can correct the impairment of lipolysis caused by obesity. A study found that the phosphorylation of proteins such as HSL without significantly altering the intracellular cAMP concentration, thereby increasing lipolysis and thermogenesis to combat obesity [111]. Therefore, in studies or models of obesity, the concentration and expression levels of cAMP are influenced by various factors. The first two studies on the changes in cAMP levels in obese individuals have contradictory conclusions, which may be related to differences in tissues and fluids, the spatiotemporal specificity of cAMP action, and the dynamic changes in the stages of obesity development. In the former study, the cAMP levels in the plasma of patients with obesity were elevated owing to factors such as immune cell activation and compensatory stress hormones, reflecting systemic inflammation and metabolic disorders. In the latter study, within WAT in the obese state, local cAMP levels were reduced owing to impairment of the β-adrenergic receptor – cAMP – PKA – HSL lipolytic pathway (such as downregulation of β-adrenergic receptor expression and decreased AC activity), leading to impaired lipolytic function and exacerbated fat accumulation. Both studies examined the role of cAMP in obesity from the perspectives of systemic compensation and local dysfunction. By inhibiting cAMP-degrading enzymes, PDE can increase intracellular cAMP levels, thereby improving obesity and related metabolic disorders. On the one hand, it promotes fat oxidation and thermogenesis by activating AMPK (for example, ginsenoside Rd enhances adaptive thermogenesis in a cAMP-dependent manner) [112]. On the other hand, it reduces the release of inflammatory factors and improves insulin sensitivity by inhibiting inflammatory signalling pathways such as those that suppress cytokine signalling 3 [113].

Thermogenesis in adipose tissue is considered a new therapeutic target for promoting energy metabolism in obesity and metabolic diseases. The effects of cAMP on the browning of BAT and WAT vary depending on the state of adipocyte differentiation and the downstream effector (PKA or EPAC) [114]. During the browning process of brown preadipocytes and WAT, cAMP-dependent EPAC promotes cell proliferation and regulates the differentiation of precursor cells into thermogenic adipocytes (beige fat), a process independent of PKA [115]. In mature brown adipocytes, cAMP enhances thermogenesis by activating PKA, inducing UCP1 expression, and promoting mitochondrial uncoupling [116]. By inhibiting PKA activity in the GABAergic neurons of the dorsal median hypothalamus, WAT browning can be induced, thereby reducing body weight and fat pad weight [117]. PKA is an important molecule involved in energy metabolism and a key molecule connecting carbohydrate and lipid metabolism. Multiple studies have shown that in obesity research model groups, PKA participates in insulin resistance, lipolysis, and BAT thermogenesis by regulating metabolic enzymes and downstream molecules [118–120]. These findings provide a reference for interventions for obesity. Furthermore, leptin, as a core secretory factor of adipocytes and a key signalling molecule in the hypothalamic ‘energy regulation center’, plays an irreplaceable role in energy metabolism homoeostasis [121]. The EPAC signalling pathway (particularly the EPAC1 subtype) is a central node in the adipocyte leptin regulatory network, functioning through direct regulation of leptin synthesis and secretion, as well as indirect modulation of leptin signal transduction via multi-layered mechanisms. This regulation exhibits significant adipocyte subtype specificity and tissue localization heterogeneity. In peripheral adipose tissue, EPAC1 clearly promotes leptin synthesis and secretion, with its regulatory activity being significantly higher in BAT and beige adipocytes compared to WAT [115]. Mechanistic studies have confirmed that treating brown adipose precursor cells with an EPAC1-specific activator (such as 8-pCPT-2’-O-Me-cAMP) activates the downstream mTORC1 signaling pathway [87,115]. This not only promotes cell proliferation and upregulates the expression of thermogenic genes Ucp1 and Pgc1a but also significantly enhances leptin gene transcription and protein secretion [87,115]. In white adipose tissue, EPAC1 activates the Rap1-PI3K-AKT signaling pathway, directly promoting leptin secretion on one hand, and enhancing adipocyte sensitivity to leptin while inducing white adipose browning on the other, thereby indirectly amplifying leptin’s energy regulatory function [87]. In contrast, within the central nervous system (e.g. the hypothalamus), the EPAC signalling pathway exerts an opposite regulatory effect on the leptin pathway. Elevated cAMP levels can activate EPAC (rather than PKA), inhibiting the leptin-activated signalling cascade in the hypothalamus and attenuating leptin-induced depolarization of pro-opiomelanocortin neurons. This ultimately weakens leptin’s anorexigenic effect and induces leptin resistance [122]. The EPAC2A subtype plays a particularly critical role in this process: EPAC2A knockout mice fed a high-fat diet showed significantly reduced defects in hypothalamic leptin signalling, enhanced suppression of food intake in response to leptin, and a markedly lower incidence of obesity [102]. Conversely, central overactivation of EPAC2A exacerbates leptin resistance, becoming a key driver of obesity. It is noteworthy that the central regulation by EPAC1 is complex. While EPAC1-deficient mice exhibit enhanced leptin sensitivity, reduced body fat, and improved glucose homoeostasis [89], another study revealed that EPAC1-deficient models did not show significant changes in food intake or circulating leptin levels. Instead, they developed β-cell dysfunction and metabolic syndrome phenotypes [123]. This suggests that the central regulation of the leptin pathway by EPAC1 May depend on dynamic changes in physiological and pathological states as well as the tissue microenvironment. Dysfunction of the EPAC-leptin axis is one of the crucial pathological mechanisms underlying obesity and related metabolic diseases. Firstly, this axis is involved in the development and progression of obesity. A study has demonstrated that under high-fat diet-induced obesity, EPAC1 deficiency triggers adipose tissue inflammation and glucose intolerance, accompanied by insufficient leptin secretion and leptin resistance [124]. Conversely, excessive activation of central EPAC2A further exacerbates leptin resistance. In EPAC2A knockout mice, high-fat diet-induced hypothalamic leptin signalling defects are significantly alleviated, and the animals exhibit enhanced responsiveness to leptin-induced feeding suppression, with a marked reduction in obesity incidence [102]. These findings indicate that the combined effects of EPAC1 insufficiency and EPAC2A overactivation disrupt the balance of the EPAC-leptin axis, ultimately contributing to the development of obesity. In terms of metabolic syndrome, activation of EPAC1 can improve insulin resistance and glucose homoeostasis in obese mice by promoting leptin secretion and enhancing leptin sensitivity. Meanwhile, it reduces the release of inflammatory factors (e.g. IL-1β) in adipose tissue, thereby alleviating metabolic disorders [125]. Additionally, obesity is often associated with a series of cardiovascular diseases such as hypertension, and leptin serves as a key mediator linking obesity to hypertension, primarily participating in blood pressure regulation through central sympathetic nerve activation [126]. In the state of leptin resistance, the metabolic regulatory function of leptin is impaired, but its sympathetic nerve activation effect remains intact, which may indirectly promote the development of obesity-related hypertension [127]. Notably, EPAC1 activators help reduce the risk of cardiovascular diseases by decreasing body weight and improving leptin sensitivity. Therefore, activating EPAC1 or inhibiting central EPAC2A may represent promising therapeutic strategies for obesity and related metabolic diseases. In addition to leptin, other cytokines also play important roles in obesity. Adiponectin is a key adipokine secreted by adipose tissue, and its levels are significantly negatively correlated with the degree of obesity. It exerts protective effects against obesity and related metabolic disorders through mechanisms such as improving insulin sensitivity, promoting lipid metabolism, and inhibiting inflammatory responses [128]. Research indicates that in white adipocytes, cAMP stimulates the rapid exocytosis of adiponectin-containing vesicles in the readily releasable pool by activating EPAC, independent of Ca^2+^ and ATP levels, achieving basal secretion levels [96]. The synergistic effect of Ca^2+^ and ATP enhances vesicle fusion efficiency and recruits reserve vesicles to the readily releasable pool, amplifying the exocytotic effect triggered by cAMP and maintaining sustained secretion. This process has a unique signalling pathway dependency, specifically relying on cAMP – EPAC rather rather than PKA-independent cAMP stimulation of white adipocyte exocytosis and adipokine secretion, with modulation by Ca^2+^ and ATP. In the state of obesity, this mechanism is disrupted owing to impaired cAMP – EPAC pathways, Ca^2+^ signal dysregulation, or insufficient ATP production, leading to reduced adiponectin secretion and further exacerbation of metabolic abnormalities. Additionally, other adipokines play key roles in obesity-related chronic low-grade inflammation and insulin resistance [129]. A study has indicated that in high-fat diet-fed animal models, increased cAMP not only improves plasma glucose levels and insulin sensitivity but also increases the levels of leptin, adiponectin, interleukin (IL)-6, and tumour necrosis factor-alpha (TNF-α) in the plasma [20,112,130].

In summary, the cAMP signalling pathway involved in obesity affects mainly lipolysis, thermogenic function, and glucose – lipid metabolism in adipose tissue. Tissue-specific regulation (such as differential activation of EPAC/PKA) and downstream effects (such as adiponectin secretion, inflammatory factor regulation, and enhanced thermogenesis) provide a multitarget intervention approach for obesity treatment, whereas the development of PDE inhibitors holds promise as a new direction for improving metabolic disorders.

### 5.2. Insulin resistance and diabetes

A complex two-way regulatory relationship exists between diabetes and lipid metabolism disorders, forming a vicious cycle. When glucose homoeostasis is disrupted, the states of hyperglycaemia and insulin resistance directly disrupt the normal lipid metabolism process, and the imbalance of lipid homoeostasis further exacerbates the progression of diabetes and the occurrence and development of its complications [131]. In this process, the cAMP – PKA/EPAC signalling pathway plays a central regulatory role. Studies over the past few decades have shown that the cAMP – PKA/EPAC pathway plays a crucial role in β-cell function and has a bidirectional regulatory effect on β-cell function. First, cAMP maintains glucose homoeostasis by amplifying glucose-stimulated insulin secretion and promoting β-cell adaptation [132]. PKA and EPAC regulate insulin secretion through multiple mechanisms, including membrane depolarization, increased Ca^2+^ sensitivity, regulation of gene expression, activation of small G proteins, regulation of ATP-sensitive potassium channels, L-type voltage-gated calcium channels, and other non-selective cation channels [133–135]. Additionally, EPAC promotes reorganization of the actin cytoskeleton by activating the small G protein Rap1, optimizing the dynamics of insulin secretion [124]. Moreover, the cAMP – PKA/EPAC pathway interacts with noncanonical pathways such as iPLA2β and C1ql3 to jointly regulate insulin secretion [133]. cAMP also regulates the survival and adaptability of β-cells through PKA/EPAC. cAMP can promote insulin secretion by inducing the expression of the transcription factor MafA, whereas the downregulation of cAMP in β-cells reduces glucose tolerance and leads to severe β-apoptosis, which may explain why increasing the intracellular cAMP level helps 3T3-L1 cells take up glucose [136,137]. Healthy islet function is a prerequisite for ensuring glucose homoeostasis. The signalling pathway mediated by PKA/EPAC/CREB/IRS-2 can promote insulin secretion, improve islet cell function and proliferation, and contribute to the improvement of islet cell health in type 2 diabetes [138]. RapGEF/EPAC may maintain the normal function of islet cells by improving autophagy function [139]. Additionally, cAMP is involved in regulating the effects of other hormones, such as regulating the secretion of glucagon, which is crucial for blood glucose balance. According to a study, some bioactive ingredients in the diet can play a positive role in the prevention and treatment of diabetes by regulating the cAMP pathway [140]. As a downstream signal responder of cAMP – PKA, CREB can also play a role in lowering blood sugar through this pathway [141]. The activation of Takeda GPCR5 enhances the conversion from glucagon to glucagon-like peptide-1 (GLP-1) synthesis in human and mouse pancreatic islet α-cells through the GS – cAMP – PKA – CREB-dependent activation of PC1. Moreover, the release of GLP-1 from α-cells induced by Takeda GPRC5 occurs through an EPAC-mediated PKA-independent mechanism [142]. In animal experiments, astragaloside IV has been shown to have an effective therapeutic effect on gestational diabetes mellitus in mice by regulating the accumulation of cAMP and hepatic gluconeogenesis [143]. Diabetes and lipid homoeostasis influence each other. The cAMP – PKA/EPAC pathway maintains blood glucose homoeostasis through multiple mechanisms, such as regulating insulin secretion in β-cells, autophagy, and gene expression. Its abnormality is closely related to the progression of diabetes. Bioactive ingredients in the diet and targeted drugs can play roles in prevention and treatment by regulating this pathway.

Diabetes can induce multiorgan lesions, and the cAMP – PKA/EPAC pathway also plays an important role in diabetic complications. In type 1 diabetes-induced diabetic cardiomyopathy, the protein level of the cAMP effector EPAC2 remains unchanged during the progression of diabetes, but the mRNA levels of EPAC1 and EPAC2 are upregulated in the diabetic myocardium. This may be related to the differential and time-specific changes in the sarcoplasmic reticulum calcium pump (SERCA2a), the phospholipid layer, and cardiac myosin (TnI) in the diabetic myocardium [144]. cAMP has potential therapeutic value in diabetic retinopathy by maintaining the retinal vascular barrier (which regulates the tight junction protein Zonula Occludens-1), inhibiting the release of vascular endothelial growth factor (VEGF), and suppressing microglial activation [145]. The 5-hydroxytryptamine/cAMP/PKA signalling pathway has potential therapeutic effects in the treatment of diabetes-related gastroparesis, providing a new therapeutic perspective for clinical practice [146]. However, further research is needed for verification. The cAMP – PKA signalling pathway is involved in regulating the function of spinal hyperpolarization-activated cyclic nucleotide-gated (HCN) channels in diabetic neuropathic pain [147]. Inhibiting PKA or HCN channels can significantly relieve the pain behaviour of diabetic rats, which provides ideas for the treatment of diabetic neuropathic pain. In summary, the cAMP – PKA/EPAC pathway plays a key role in the multiple organ complications of diabetes, providing multidimensional targets for the treatment of complications.

Future research should focus on the molecular mechanisms by which cAMP – PKA/EPAC precisely and dynamically regulates insulin secretion, as well as the survival and adaptability of β-cells in different metabolic microenvironments, and the development of highly selective regulators targeting the PKA/EPAC pathway. Additionally, exploring combined intervention strategies involving the PKA/EPAC pathway with other key metabolic pathways is necessary to overcome the vicious cycle between diabetes and lipid metabolism disorders and to develop tissue-specific delivery systems to achieve precise targeting of key tissues, such as the liver and islets, by drugs, thereby enhancing the therapeutic effect and reducing side effects.

### 5.3. Fatty liver

Ectopic deposition of fat in the liver caused by abnormal lipid metabolism is the main cause of fatty liver. Disease-inducing factors can be classified into two major categories: alcoholic and non-alcoholic fatty liver [148]. The most common inducing factors of non-alcoholic fatty liver disease (NAFLD) include obesity and insulin resistance. The cAMP – PKA pathway is crucial for the regulation of lipid metabolism and is closely related to the progression of NAFLD. An increase in the intracellular cAMP level leads to the activation of PKA, which inhibits the transcriptional activity of genes related to lipid metabolism, reduces the expression of fat-related genes, and enhances lipolysis, thereby reducing lipid accumulation in the liver. Moreover, PKA can regulate the activity of PPARα through phosphorylation. Additionally, PPARα is expressed mainly in the liver and is an important transcription factor for regulating lipid and glucose homoeostasis. The overexpression or activation of PKA can alleviate oxidative stress, reduce lipid deposition, improve insulin resistance, and correct metabolic disorders [149]. The role of cAMP – PKA in fatty liver is reflected mainly in its association with the regulation of endoplasmic reticulum stress and iron homoeostasis [150]. cAMP-dependent PKA phosphorylates inositol-requiring enzyme 1 (IRE1), which is activated in non-alcoholic fatty liver and participates in the endoplasmic reticulum stress response of hepatocytes. This response further affects iron metabolism and lipid peroxidation, thus exacerbating ferroptosis. Therefore, the cAMP/PKA/IRE1 pathway forms a vicious cycle in the pathological progression of non-alcoholic fatty liver through the ‘ER stress – iron metabolism disorder – ferroptosis’ axis. This is different from the traditional view that PKA affects the progression of fatty liver by regulating metabolic enzymes. This study revealed that in the context of non-alcoholic fatty liver disease, the phosphorylation of IRE1 by PKA can specifically increase endoplasmic reticulum stress, suggesting that the regulatory effect of PKA on fatty liver disease may be partially dependent on the cellular environment. Additionally, CREB can participate in the progression of fatty liver diseases by regulating lipid β-oxidation and inhibiting lipid synthesis and fibrosis [151]. The antifibrotic effect of CREB can also be achieved by downregulating the KCa ion channel through the EPAC pathway [152]. Moreover, the N-glycosylation of CREBH improves lipid metabolism and attenuates lipotoxicity by regulating PPARα and stearoyl-CoA desaturase 1 [153]. The CREB-Pgc1α pathway affects metabolic changes under a high-fat diet by regulating the interaction between lipid droplets and mitochondria (such as activating ATGL/Plin5 and depending on the Rab32 protein) [154]. Additionally, cholestasis can inhibit lipid synthesis through the AMPK/CREB pathway, and chronic noise exposure activates the CRTC2/CREB and SREBP1/SCD pathways via the gut – liver axis, leading to disorders of glucose and lipid metabolism [155]. These findings provide multidimensional targets for mechanistic analysis and intervention strategies for fatty liver disease.

### 5.4. Cardiovascular diseases

Cardiovascular diseases are a major cause of death in the global population. According to statistics, compared with that in 2025, the prevalence of cardiovascular diseases is expected to increase by 90.0%, and crude mortality is expected to increase by 73.4% by 2050 [156]. The cAMP – PKA/EPAC signalling pathway also plays an important role in the onset and progression of cardiovascular diseases. In the cardiovascular system, under normal physiological conditions, lipids in the blood (such as cholesterol and triglycerides) combine with apolipoproteins to form lipoproteins, which are transported in the blood in the form of lipoproteins. When lipid metabolism is disrupted, the levels of low-density lipoprotein (LDL), particularly oxidized LDL (ox-LDL), in the blood increase. These ox-LDLs are easily phagocytosed by monocytes. After monocytes phagocytose ox-LDL, foam cells are formed and deposited under the arterial intima, gradually forming lipid streaks, which ultimately induce atherosclerosis. Research has indicated that cAMP/EPAC1 signalling plays a fundamental role in vascular remodelling during the development of atherosclerotic lesions by promoting ox-LDL uptake and foam cell formation [157]. Ox-LDL stimulates bone marrow-derived macrophages, leading to increased intracellular cAMP and EPAC1 levels. EPAC1 can also upregulate ox-LDL receptor 1 by activating protein kinase C, promoting the uptake of ox-LDL and thereby driving the formation of foam cells and the progression of atherosclerosis [157]. Therefore, EPAC1 is a promising new target for the treatment of atherosclerosis. Many cardiovascular diseases are induced by signals mediated by GPCRs through the G protein-stimulating α subunit (Gsα) protein. In the macrophages of human and mouse plaques, Gsα and active Gsα are significantly upregulated. Ox-LDL can cause the translocation of Gsα in the lipid rafts of macrophages in the short term, and in the long term, it can promote Gnas transcription to increase Gsα expression by activating ERK1/2 and C/EBPβ phosphorylation through oxidative stress. During the process of atherosclerosis, Gsα enhances macrophage lipid uptake and foam cell formation through the cAMP/CREB pathway [158]. Blocking the Gsα–cAMP pathway (such as the suramin pathway) or regulating CREB activity may inhibit abnormal cardiovascular lipid metabolism. Myocardial infarction is another disease with extremely high mortality in addition to atherosclerosis. During myocardial infarction, the role of the cAMP – PKA pathway is complex. First, the activation of PKA before myocardial infarction ischaemia can limit infarct size by inhibiting Rho-kinase. Additionally, the antioxidant N-acetylcysteine activates glutathione, promotes cAMP production and AC-induced PKA activity, subsequently inhibits glycogen synthase kinase 3β, prevents connexin 43 internalization, and exerts an antiarrhythmic effect. Reports have shown that adrenomedullin, when activated through this signalling pathway, can limit infarct size and promote the opening of mitoKCa channels. Under myocardial stress, beta-adrenergic receptor signalling can increase PKA activity to counteract cardiac dysfunction. Moreover, the activation of PKA by this signalling pathway can promote the efflux of calcium ions, reduce calcium overload, decrease cell injury and necrosis, maintain the metabolism and membrane stability of cardiomyocytes, and increase survival [159]. In contrast, excessive activation of the cAMP – PKA signalling pathway can increase the excitability of cardiomyocytes, induce arrhythmia, and promote fibrosis in cardiac tissue [160]. Therefore, the results of different studies are contradictory. Overall, the cAMP – PKA signalling pathway plays an important protective role in myocardial infarction, but moderate activation of this pathway is beneficial for reducing the degree of cardiomyocyte apoptosis, promoting survival, and enhancing cardiac function, whereas excessive activation may induce arrhythmia [161]. During the process of myocardial fibrosis after myocardial infarction, the cAMP signalling pathway has a dual regulatory effect on the expression of connective tissue growth factor. cAMP can upregulate the expression of PKA through the p44/42MAPK pathway and simultaneously reduce the phosphorylation level of p44/42MAPK, followed by the inhibition of the profibrotic effect of connective tissue growth factor [162]. Although the short-term increase in cAMP levels after myocardial infarction can compensate for the increase in cardiac contractile function, long-term continuous activation can induce cardiac remodelling and pathological fibrosis [159]. Therefore, targeting the cAMP signalling pathway (such as regulating PKA activity or p44/42MAPK phosphorylation) is expected to become a new strategy to inhibit myocardial fibrosis, reduce arrhythmia, and protect myocardial function. After myocardial infarction in patients with diabetes, the function of the cAMP – PKA signalling pathway is inhibited, mainly through the inhibition of the β-adrenergic pathway, which generates cAMP. In particular, under high-glucose conditions, the cAMP – PKA signalling pathway and its upstream β-adrenergic pathway are more vulnerable to damage, thus affecting the activity of the downstream brain-derived neurotrophic factor/tropomyosin receptor kinase signalling pathway and hindering the repair of myocardial ischaemia – reperfusion injury [163]. Adrenoceptor beta 2 agonists can promote the activity of the brain-derived neurotrophic factor/tropomyosin receptor kinase and cAMP – PKA signalling pathways and play a protective role in diabetic cardiac ischaemia – reperfusion injury [163]. Additionally, by activating the adenosine A2a receptor, Shenfu injection inhibits the continuous activation of cAMP, reduces collagen synthesis and matrix metalloproteinase-9 expression, and alleviates fibrosis after myocardial ischaemia – reperfusion [164]. In obesity and type 2 diabetes models, a significant β2-AR-stimulated cAMP response exists within the PLN/SERCA2a domain, as does a desensitization response to cAMP under β1-AR stimulation. This desensitization response may be due to the loss of the local cAMP-degrading enzyme PDE4, thus forming an internal compensatory mechanism to maintain calcium ion dynamics and smooth cardiac diastolic function [165]. A similar study revealed that in heart failure related to obesity and type 2 diabetes, variations in cAMP microdomain signalling play important roles in cardiac function [166]. Additionally, cAMP can also participate in the occurrence of cardiovascular diseases through various mechanisms. cAMP – PKA signalling can exacerbate myocardial hypertrophy through autophagy [167]. A high-fat diet inhibits the cAMP signalling pathway in rat renal tissue, leading to a decrease in histone deacetylases (such as sirtuin 1) and an increase in P300 expression. Then, through histone H3K27 acetylation, it upregulates angiotensin-converting enzyme 1, promoting vascular remodelling and hypertension [168]. In conclusion, the cAMP – PKA pathway has broad application prospects in the field of cardiovascular lipid homoeostasis.

As a key response protein in the cAMP signalling pathway, EPAC is deeply involved in the occurrence and development of cardiovascular diseases. The saturated fatty acid palmitate can cause palmitoylation of sAC at the highly conserved Cys342 residue, thereby stimulating cAMP production and activating EPAC1 [100]. After activation, EPAC1 enhances the activity of CPT-1, accelerating the uptake and storage of fatty acids by mitochondria. It phosphorylates key enzymes involved in fatty acid β-oxidation, such as long-chain fatty acyl-CoA dehydrogenase and 3-ketoacyl-CoA thiolase, through the Ca^2+^/CAM-dependent protein kinase II (CaMKII)-dependent pathway, inhibiting fatty acid catabolism. Additionally, the EPAC1–CaMKII axis can interact with the α subunit of ATP synthase to regulate mitochondrial energy metabolism [89]. Together, these effects disrupt the balance between mitochondrial fatty acid uptake and oxidation, leading to lipid accumulation, mitochondrial dysfunction, and cardiomyocyte death, ultimately disrupting lipid homoeostasis. Rap1, a small GTP-binding protein in the Ras superfamily, plays multiple roles in cardiovascular biological processes. Pharmacological activation of the EPAC – Rap1 pathway can significantly increase the survival, adhesion, and differentiation capabilities of transplanted mesenchymal stem cells (MSCs), promoting functional repair in the myocardial infarction area [169]. Vitexin reduces reactive oxygen species levels, stabilizes the mitochondrial membrane potential, and inhibits both cytochrome c release and Bax recruitment to mitochondria through the EPAC1–Rap1 signalling pathway [170]. These two studies explored the repair and protective effects of EPAC1–Rap1 in the fields of cell therapy and natural medicine on the myocardium, providing a theoretical basis for the use of EPAC as a therapeutic target. Additionally, GLP-1 receptor agonists exert antioxidant, antiapoptotic, and mitochondrial protective effects in cardiomyocytes by activating the cAMP – EPAC signalling pathway. The therapeutic efficacy of these compounds can be further optimized by regulating cAMP metabolism (such as by inhibiting PDE-4). These findings provide new therapeutic strategies targeting the GLP-1 receptor/cAMP – EPAC axis for cardiovascular metabolic diseases, such as diabetic cardiomyopathy and heart failure [10,171]. Heart failure induces left atrial-specific fibrosis, promotes atrial fibrillation, and activates the adrenergic system. This process promotes remodelling through various signalling molecules, including cAMP – EPAC. EPAC1 signalling plays a crucial role in preventing profibrotic cardiac remodelling [172]. When heart failure occurs, the activation of Epac1 regulates the function of atrial fibroblasts, promotes collagen synthesis and extracellular matrix remodelling, exacerbates left atrial fibrosis and electrophysiological abnormalities, and ultimately induces atrial fibrillation. Therefore, inhibiting EPAC1 is expected to become a therapeutic target for preventing profibrotic cardiac remodelling. Additionally, a research has shown that a high-fat diet significantly reduces the infarct size in wild-type and EPAC2-deficient mice but has no obvious effect on EPAC1-deficient mice [173]. These findings reveal that the EPAC1-dependent signalling pathway plays an important role in the cardioprotection induced by a high-fat diet, which may be related to the ‘obesity paradox’, that is, obesity is associated with lower mortality and incidence of cardiovascular diseases.

In conclusion, these research results support the therapeutic and developmental prospects of EPAC and its signalling pathway in cardiovascular diseases. Although the roles of cAMP – PKA/EPAC in lipid homoeostasis-related cardiovascular diseases are mostly in the basic research stage, these findings indicate that this signalling pathway has certain preventive and therapeutic value. A future challenge may lie in how to precisely and synergistically regulate this signalling pathway to ensure that it functions as expected across various cardiovascular disease complications.

## 6. cAMP signaling in the adipose microenvironment

### 6.1. cAMP and immune cells

cAMP and its signalling pathway, through complex two-way crosstalk between adipocytes and immune cells in adipose tissue, form the immune microenvironment of adipose tissue and are deeply involved in lipid metabolism, inflammation, and the insulin response.

#### 6.1.1. Macrophages

In the microenvironment of adipose tissue, multiple signalling pathways are intertwined and jointly regulate immune – metabolic homoeostasis. Among them, the cAMP signalling pathway, as a key regulatory hub, has received significant attention in recent years. cAMP not only participates in the metabolic regulation of adipocytes and maintenance of energy balance but also plays an important role in the regulation of immune cell functions. As the core immune cell population in the adipose tissue microenvironment, macrophages are closely related to pathological processes such as chronic inflammation and insulin resistance induced by obesity.

*cAMP and macrophages*: A study found that the activation of the Gsα/cAMP/CREB signalling axis promotes lipid uptake, inhibits lipid efflux, and affects the inflammatory response, thus exacerbating the formation of atherosclerotic plaques [158]. One research indicates that under the induction of ox-LDL, the Ca^2+^ level in macrophages increases, subsequently activating calpains. These calpains stimulate AC through the activation of Gsα, thus increasing the concentration of cAMP. The increase in cAMP further enhances the production of inositol trisphosphate (IP3) through Rap2B- and phospholipase Cε-dependent mechanisms. IP3 then induces the release of Ca^2+^ from the endoplasmic reticulum via the IP3R receptor, and this feedback mechanism inhibits autophagy. By using the calcium chelator BAPTA-AM and the calpain inhibitor calpeptin, the levels of Ca^2+^, calpain 1, calpain 2, and downstream effectors (Gsα, cAMP, and IP3) can be reduced, thereby enhancing autophagic function, improving the antilipid capacity of macrophages, inhibiting foam cell formation, and ultimately suppressing atherosclerotic plaque development [174]. Additionally, the contribution of cAMP activation to atherosclerosis may occur through another mechanism. In macrophages, cAMP significantly upregulates the expression of the ox-LDL receptor LOX-1 by activating its downstream effector molecule EPAC1 (rather than PKA), thereby promoting the uptake and intracellular accumulation of oxidized LDL, ultimately leading to the formation of foam cells and the development of atherosclerosis [157]. The specific mechanism involves EPAC1 enhancing the expression of LOX-1 by activating the Rap1 signalling pathway or regulating transcription factors such as nuclear factor-kappa B (NF-κB), disrupting the balance between lipid uptake (such as through LOX-1, SR-A, and CD36) and cholesterol efflux (such as through ABCA1/ABCG1) in macrophages and causing cells to accumulate lipids. This discovery not only reveals the crucial role of the cAMP/EPAC1/LOX-1 axis in atherosclerosis but also provides new therapeutic ideas for targeted intervention in the EPAC1 signalling pathway to alleviate atherosclerosis.

*cAMP – PKA and macrophages*: In the microenvironment of adipose tissue, the cAMP – PKA signalling pathway plays a central role in metabolic homoeostasis and inflammatory responses by regulating the polarization and function of macrophages. Various metabolites and hormones (such as taurocholic acid, L-lactic acid, EETs, and abscisic acid) activate cAMP signalling through different receptors (such as GPR132 and PPARγ), thereby influencing the phenotypic transformation of macrophages and the metabolism of adipose tissue [175–178].

Numerous studies have shown that promoting M2 anti-inflammatory polarization and adipose browning is highly important for improving metabolic problems such as obesity-related inflammation and insulin resistance [175,179,180]. Shengmai San promotes the secretion of slit homolog 3 protein by M2 macrophages through the gut microbiota metabolite taurocholic acid [175]. The Slit homolog 3 protein activates the PKA/CaMKII pathway in sympathetic neurons through Roundabout receptor 1, promotes the release of norepinephrine, and then activates the cAMP/PKA/pHSL pathway in adipocytes, inducing adipose browning and improving obesity [175]. The deletion of fatty acid synthase in adipocytes mimics cold signals and activates the immunometabolic response of macrophages. Adipocytes lacking this enzyme upregulate chemokines such as C-C motif chemokine ligand 2 and colony stimulating factor 1, recruit macrophages to WAT, and significantly increase M2 polarization markers, which is related to the activation of the PPARγ/signal transducer and activator of transcription 6 signalling pathway. By secreting catecholamines (such as norepinephrine) or IL-4/IL-13, macrophages can also promote the browning of adipocytes in a reverse manner [179]. In obesity, a reduction in catecholamine resistance can drive the secretion of IL-6 by adipocytes in response to catabolic signals. By restoring the sensitivity of adipocytes to catecholamines, amlexanox stimulates the secretion of adipocyte-specific IL-6, thereby activating local macrophage signal transducer and activator of transcription 3, promoting the expression of IL-4 receptor α, making macrophages sensitive to IL-4 signals, and promoting the expression pattern of anti-inflammatory genes and M2 polarization [181]. Additionally, exosomes derived from M2-type macrophages can promote the differentiation of fibroadipogenic progenitors into beige adipocytes in vitro [182]. These findings suggest that M2-type macrophages may regulate the phenotypic conversion of adipocytes through bioactive molecules (such as specific miRNAs and protein factors) delivered by exosomes, thereby participating in the regulation of energy metabolism balance (e.g. enhancing thermogenesis and improving insulin sensitivity). These findings provide new insights into the pathogenesis of obesity and metabolic diseases (such as type 2 diabetes) and indicate that targeting the M2 macrophage – exosome – fibroadipogenic progenitor axis may serve as a potential intervention strategy to promote beige adipogenesis and improve metabolic health.

Multiple studies have shown that inhibiting M1 proinflammatory polarization helps improve insulin resistance [176,177]. L-lactic acid activates cAMP – PKA signalling through the GPR132–Gs protein, further inhibiting the polarization of M1 macrophages, reducing the release of proinflammatory factors, and alleviating obesity-related insulin resistance [176]. EETs inhibit the transformation of macrophages into the M1 phenotype through the cAMP – EPAC pathway, maintain the M2 phenotype, reduce adipose tissue inflammation, and increase insulin sensitivity [177]. The cAMP signalling axis can also coordinately regulate lipid metabolism and inflammation. For example, abscisic acid and rosiglitazone synergistically activate the cAMP/PKA/PPARγ axis, reduce the infiltration of adipose tissue macrophages, and improve glucose tolerance and lipid metabolism disorders [178]. The deletion of G protein signalling modulator 1 upregulates the transcription of the TNFAIP3 gene through the Gαi3/cAMP/PKA/CREB axis and inhibits Toll-like receptor 4 (TLR4)-induced NF-κB signalling, thereby reducing the inflammatory response [183]. This regulatory mechanism can not only alleviate local inflammation in adipose tissue but also improve systemic metabolic disorders such as insulin resistance associated with obesity and type 2 diabetes. Moreover, the relationship between body mass index and inflammation is regulated by cell types such as regulatory T cells (Tregs) and macrophages in adipose tissue [178]. These findings indicate that the immune microenvironment of adipose tissue affects overall lipid homoeostasis in the body in multiple ways.

*cAMP – EPAC and macrophages*: The cAMP – EPAC pathway plays a crucial regulatory role in the regulation of M1/M2 polarization. Numerous studies have shown that the activation of M1 macrophages is involved in adipose tissue inflammation [184,185]. In obesity, the infiltration of M1 macrophages in visceral adipose tissue increases, and the secretion of proinflammatory factors such as TNF-α may be regulated by the cAMP/EPAC – ST2 pathway [186]. The activation of EPAC2 is positively correlated with the expression of ST2, suggesting that macrophages participate in adipose tissue inflammation and fibrosis through this pathway [186]. Additionally, endogenous prostaglandin E2 (PGE2) increases cAMP levels through EP2/EP4 receptors and activates EPAC2 and NF-κB, thereby promoting IL-33 production in macrophages and increasing inflammation in adipose tissue [186]. cAMP may regulate macrophage polarization by inhibiting PKA or activating EPAC. The activation of EPAC2 May favour the M1 phenotype, and the inhibition of PKA may reduce the differentiation of M2 anti-inflammatory macrophages, jointly exacerbating the inflammatory microenvironment of adipose tissue [187]. Moreover, M1 macrophages inhibit the activity of lipolytic enzymes (such as ATGL) by secreting TNF-α, thereby disrupting lipid storage homoeostasis; activation of the EPAC2–ST2 pathway may further reinforce this process, which is associated with obesity-related metabolic disorders [188,189]. Deletion of EPAC1 reduces the infiltration of M1 macrophages and improves insulin resistance in obese mice.

In conclusion, the cAMP – PKA/EPAC signalling pathway plays a central role in the regulation of macrophage polarization and maintenance of lipid homoeostasis. By regulating the differentiation of macrophages towards the anti-inflammatory (M2) or proinflammatory (M1) phenotype and influencing the metabolic and storage processes of adipocytes, it profoundly affects the metabolic balance of the body.

#### 6.1.2. T cells

*In variant natural killer T (iNKT) cells*: The cAMP signalling pathway and iNKT cells form a complex metabolic – immune regulatory network through downstream molecules in the immune microenvironment of adipose tissue [190]. Research has shown that iNKT cells exhibit specific metabolic characteristics according to the tissue environment they are in. iNKT cells in adipose tissue maintain tissue homoeostasis through downstream molecules of cAMP, such as AMPK, and play a role in obesity-induced inflammation [190]. Additionally, iNKT cells in adipose tissue display distinct tissue-specific phenotypes under basal conditions, including high levels of lipid storage and increased production of interferon-γ when exposed to exogenous lipids. Lipid accumulation in adipose tissue-resident iNKT cells contributes to an inflammatory phenotype. These cells are highly sensitive to the lipid environment, can absorb and store environmental lipids more rapidly, and have greater reactivity than other immune cell populations do. Research also indicates that the immunometabolic regulation of iNKT cells largely depends on their adaptability to the lipid environment. Moreover, compared with the CD3+ immune cell population, iNKT cells residing in adipose tissue are highly responsive to the lipid environment, which enhances their function in adipose tissue-specific lipid accumulation in adipose tissue-resident iNKT cells, contributing to an inflammatory phenotype. In the immune microenvironment of adipose tissue, the cAMP signalling pathway has a significant effect on iNKT cells. iNKT cells are abundant in WAT and can affect metabolic functions by regulating immune responses. Adipocytes directly activate iNKT cells through CD1d-mediated lipid antigen presentation, and this interaction is also associated with adipokines such as leptin. Leptin inhibits the function of iNKT cells through the MAPK pathway, and the disruption of leptin signalling exacerbates metabolic disorders such as insulin resistance. The imbalance of this regulatory network may lead to metabolic diseases, suggesting that targeting the interaction between the cAMP signalling pathway and iNKT cells could be a new strategy for treating metabolic diseases related to obesity [191]. Therefore, the imbalance of this regulatory mechanism may lead to metabolic diseases, indicating that targeting the interaction between the cAMP signalling pathway and iNKT cells could be an optional treatment for related metabolic diseases (such as obesity). This regulatory mechanism has a potential synergistic effect with the cAMP – PKA signalling pathway mediated by T-cell death-associated gene 8 (TDAG8). As a proton-sensitive GPCR, TDAG8 can activate the cAMP – PKA pathway in an acidic microenvironment (such as adipose tissue inflammation associated with obesity), promoting the phenotypic transformation and migration of vascular smooth muscle cells and accelerating the progression of atherosclerosis. These findings indicate that the cAMP signalling pathway in adipose tissue is regulated by a network of different receptors (such as TDAG8) and cell types (iNKT cells and vascular smooth muscle cells). Additionally, tissue acidification caused by metabolic disorders may affect both immune responses and vascular remodelling simultaneously through the TDAG8–cAMP axis. The subsequent inflammatory response mediated by iNKT cells and vascular lesions activated by TDAG8 May promote the development of metabolic cardiovascular diseases. Therefore, intervention strategies targeting the cAMP signalling pathway need to comprehensively consider its dual regulatory effects on immune (such as iNKT cells) and vascular cells, providing a theoretical basis for the development of new therapies that can simultaneously improve metabolic abnormalities and cardiovascular complications.

*T helper (Th) and Treg cells*: As an alarmin, IL-33 activates Th type 2 (Th2) cells through the ST2L receptor, promoting the secretion of IL-4/IL-13 and driving an anti-inflammatory response (IL-33, an IL-1-like cytokine that signals via the IL-1 receptor-related protein ST2 and induces Th2-associated cytokines) [192]. However, some studies have shown that IL-4/IL-13 secreted by Th2 cells can promote adipocyte differentiation (lipogenesis) [193,194]. These findings indicate that the IL-33/ST2L – Th2 axis has a dual role in adipose tissue. It can alleviate metabolic inflammation through anti-inflammatory effects but may also promote lipogenesis, thereby exacerbating obesity-related lipid accumulation. In addition, IL-33-induced Treg and Th2 responses in adipose tissue exhibit sex-based differences. In female mice, Th2 cells tend to promote lipogenesis, whereas in male mice, the anti-inflammatory effect is more prominent [195]. IL-33–ST2L signalling maintains metabolic balance under homoeostasis. However, this balance is disrupted by the increase in sST2 (decoy receptor) in obesity, leading to dominance of the lipogenic effect of the Th2 response [196]. Therefore, the dual role of IL-33 May be related to the tissue microenvironment, disease stage, and receptor balance. Clinical study has shown that EPAC2 May indirectly promote the Th2 response by increasing the expression of ST2L, which is positively correlated with prostaglandin E synthase-2/PGE2. However, elevated sST2 levels in obese individuals may counteract this effect [189]. Research has also shown that the activation of EPAC2 May indirectly affect lipid turnover through the function of Tregs [103,197]. In normal adipose tissue, IL-33 promotes ST2+ Tregs through cAMP – EPAC signalling, maintaining an immunosuppressive microenvironment [103]. However, animal experiments have shown the absence of Tregs in the hearts of Zucker obese rats [197]. This discrepancy may be attributed to the competitive inhibition of IL-33 signalling by sST2 or the dominance of local proinflammatory factors (such as interferon-γ).

#### 6.1.3. Other immune cells

In addition to macrophages and T cells, mast cells, eosinophils, and stromal cells in the adipose tissue microenvironment are also regulated by the cAMP signal, with effects that are cell type specific. In the context of lipid metabolism, follicle-stimulating hormone binds to its receptor on rat Sertoli cells and activates the associated signalling pathway, regulating the expression of lipid storage – related genes. This increases the intracellular lipid droplet content, helping maintain lipid storage homoeostasis and providing energy reserves or supporting other physiological processes. Follicle-stimulating hormone also regulates lipid storage in mast cells via the cAMP – PKA pathway [198]. In the regulation of immunity and fibrosis, excessive IL-33 signalling may activate mast cells or eosinophils and promote fibrosis (such as in asthma models) [199]. However, in obese tissues, due to the multifactor influence of IL-33 on the regulation of Th2 and Treg cells, the impact of IL-33/ST2 signalling on fibrosis remains controversial and needs further verification [103]. IL-33 activates eosinophils through ST2L, promotes the secretion of IL-5/IL-13 through cAMP – EPACsignal, and drives the type 2 immune response. On the one hand, it induces adipose beiging and improves metabolism. On the other hand, IL-13 activates fibroblasts, promotes collagen synthesis, and balances the improvement in metabolism and the risk of fibrosis by regulating the secretion of cAMP-dependent IL-13. In adipose stromal cells, this study revealed that human adipose tissue-derived stromal cells significantly inhibited the proliferation of human lymphocytes through indoleamine 2,3-dioxygenase activity [200]. In obesity, CD11c+ innate immune cells (mainly M1 macrophages and inflammatory dendritic cells) are recruited to adipose tissue and induce an inflammatory state, leading to insulin and catecholamine resistance. Study has shown that the cAMP signal in CD11c^+^ innate immune cells regulates systemic metabolism by controlling norepinephrine levels in adipose tissue, modulating catecholamine-induced lipolysis, and enhancing thermogenesis – ultimately promoting a lean phenotype [201]. With respect to endothelial and perivascular immune cells, studies have shown that in the epicardial adipose tissue of overweight patients with cardiovascular diseases, ST2+ and IL-33+ cells are distributed mainly around blood vessels. EP4–cAMP – EPAC2 signalling promotes perivascular inflammation and fibrosis by upregulating ST2 [197]. Additionally, the PGE2–EPAC2–ST2 axis may increase the sensitivity of endothelial cells to mechanical stress (such as hypertension), exacerbate adipose tissue hypoxia, and promote the release of proinflammatory factors [189]. These findings suggest that the cAMP signal in the adipose tissue microenvironment widely participates in the processes of metabolism, immunity, and fibrosis through the differential regulation of different cells, which is crucial for maintaining lipid homoeostasis in the body.

### 6.2. Fibroblasts

Fibroblasts, as important components of the adipose tissue microenvironment, have a complex two-way communication mechanism with adipocytes. The conditioned medium of adipocytes can induce the transformation of fibroblasts into myofibroblasts, and the application of adipose MSCs combined with basic fibroblast growth factor (bFGF) can significantly improve myocardial fibrosis [202,203]. This cell interaction profoundly affects the structural remodelling and metabolic function of adipose tissue by altering the differential expression of extracellular matrix (ECM) proteins [204]. The ECM, as the external environment for cell survival, comprises various proteins such as collagen, elastin, fibronectin, laminin, and biological macromolecules such as glycosaminoglycan, forming a complex fibrous network structure that supports and fixes cells and participates in key physiological processes such as cell signal transduction and material exchange. When fibroblasts interact with adipose lineage cells, they significantly affect the production of ECM proteins. In the early stage of adipogenesis, during the differentiation of preadipocytes into mature adipocytes, communication with fibroblasts prompts fibroblasts to secrete specific ECM proteins, such as fibronectin and certain types of collagen. These ECM proteins create a suitable microenvironment, providing physical support and biochemical signals for the proliferation, migration, and subsequent differentiation of preadipocytes. For example, fibronectin can bind to integrin receptors on the surface of preadipocytes, activating intracellular signalling pathways and thus promoting the differentiation process of preadipocytes [205]. As adipocytes gradually mature, their interaction with fibroblasts further changes. Mature adipocytes secrete adipokines such as leptin and adiponectin, which act on fibroblasts and regulate the secretion pattern of their ECM proteins [206]. Under normal physiological conditions, this regulation helps maintain the homoeostasis of the ECM in adipose tissue, ensuring that adipocytes can efficiently store and metabolize lipids. However, under pathological conditions such as obesity, the internal environment of adipose tissue changes significantly. On the one hand, the excessive accumulation of lipids causes adipocyte hypertrophy, leading to the secretion of more inflammatory factors, such as TNF-α and IL-6. These inflammatory factors stimulate fibroblasts to synthesize and secrete ECM proteins, such as type I and type III collagen, in large quantities, leading to excessive ECM deposition and fibrosis [207]. On the other hand, hypoxia – commonly present in obese adipose tissue – activates multiple profibrotic signalling pathways in fibroblasts, further exacerbating abnormal ECM production and disrupting normal interactions between adipocytes and fibroblasts [208]. Therefore, changes in the composition and structure of ECM proteins directly affect the physical properties and functional state of adipose tissue. A normal ECM structure is conducive to maintaining the morphology of adipocytes, promoting lipid metabolism, and regulating the immune response within adipose tissue. When the ECM undergoes excessive fibrosis due to dysregulation of the interaction between fibroblasts and adipose lineage cells, it disrupts the normal structure of adipose tissue, hinders the metabolic activities of adipocytes, triggers inflammatory responses, and ultimately leads to an imbalance in lipid homoeostasis. Therefore, intervening in the interaction between cells or regulating the production of ECM proteins is expected to improve the function of adipose tissue and restore lipid homoeostasis, providing new opportunities for the prevention and treatment of diseases related to lipid metabolism disorders, such as obesity and diabetes. Additionally, multiple studies have shown that fibroblasts play a role in dynamically regulating lipid homoeostasis in various disease models by secreting various growth factors (Table 1 [209–225]). FGF-binding proteins also play important roles in lipid homoeostasis. Research has indicated that in obese mice, the expression of exogenous FGF-binding protein 3 reduces hyperglycaemia, hepatic steatosis, and weight gain; inhibits de novo lipogenesis in the liver and adipose tissue; increases circulating adiponectin levels; and decreases nonesterified fatty acids [226]. Therefore, the dynamic regulation of the adipose tissue microenvironment plays a central role in lipid homoeostasis, providing multitarget intervention strategies for the treatment of metabolic diseases. Future research needs to further clarify the spatiotemporal-specific roles of different FGF subtypes and their potential for clinical transformation.

**Table 1.** Research on FGFs in lipid homoeostasis.

Growth factor	Model	Dosage	Duration	Core mechanism	Core metabolic effects	Ref
FGF-2 (bFGF)	Adipose tissue-derived stromal cells	5 ng/mL	Not mentioned	Synergizes with IL-4 to induce eotaxin-1	Regulates the inflammatory microenvironment of adipose tissue	[209]
FGF-2	5-week-old female BALB/c SIC-nu-nu mice	100 μg/mL	＞ 4 weeks	Induces adipocyte proliferatio, angiogenesi, and adipogenesis	Enhances subcutaneous adipogenesis	[210]
FGF-2	ACTA1-Cre-miR-29a fl/fl and Pax7-Cre-miR-29a fl/fl mice	Not mentioned	Not mentioned	Activates miR-29a/SPARC axis to regulate progenitor differentiation	Promotes intramuscular adipogenesis	[211]
FGF-1	Human adipose-derived mesenchymal stem cells	5 ng/mL	Not mentioned	Modulates adipogenic transcription factors (C/EBPα, PPARγ)	Enhances cell proliferation & adipogenic potential	[212]
FGF21	3-month-old male C57BL/6 mice	Not mentioned	Not mentioned	Targets signalling pathways in liver/adipose tissue	Promotes fatty acid oxidation & inhibits adipogenesis	[213]
FGF21	8-week-old C57BL/6J male mice	1 mg/kg	2 weeks	Enhances autophagy	Reverses lipid accumulation and hypertriglyceridaemia	[214]
FGF21	Islets INS-1E cells	100 nM	Not mentioned	Activates AMPK-ACC/PPAR δ/γ pathways (raises CPT1A)	Boosts fatty acid oxidation; reduces β-cell lipid deposition	[215]
FGF21	A high-fat and high-cholesterol diet APOE *3-Leiden.CETP mice	2 × 10^10^ gene (IV injection)	Once	Blocks hepatic lipid influx; inhibits macrophage activation	Prevents hepatic lipotoxicity; activates thermogenic tissues	[216]
FGF21	7-week-old male C57BL/6J mice fed with HFD	Not mentioned	Not mentioned	Activates thermogenesis in brown/beige adipose tissue	Promotes energy expenditure	[217]
FGF1	Male db/db mice of the C57BL/6J strain fed on HFD	0.5 mg/kg body weight every other day	4 weeks	Enhances adipocyte mTORC2/Rictor signalling pathway	Improves adipose tissue inflammation; alleviates systemic insulin resistance	[218]
FGF1	High-fat diet -induced mouse model ofnon-alcoholic fatty liver disease	0.5 mg/kg body weight	8 weeks	Reduces recruitment of DNA methyltransferase 3α to the IGFBP2 genomic locus	Reduces severity of insulin resistance, hyperlipidaemia, and inflammation	[219]
FGF6	C57BL/6J mice	100 ng/μL rFGF6 (75 μL/mouse, footpad injection)	2 days	Promotes adipocyte progenitor cell proliferation	Maintains adipose tissue homoeostasis	[220]
FGF6	C2C12 mice on a high-fat diet	100 ng/mL rFGF6 (cells); AAV vectors (muscle)	24 h	Activates the skeletal muscle AMPK/mTOR pathway	Anti-obesity; improves insulin sensitivity & hepatic lipid metabolism	[221]
		100 ng/mL rFGF6 (cells); AAV vectors (muscle)	Once			
FGF6	MEF line, C2C12 myoblast, and C3H/10T1/2 mouse multipotent mesenchymal progenitor cell lines	200 ng/ml	24 h, 48 h, 6 days	Regulates UCP1 via brown-adipocyte-independent transcriptional network	Modulates systemic energy metabolism	[222]
FGF9		100 ng/ml				
FGF19	LPS-induced male C57BL/6J mice	0.1 mg/kg (daily tail vein injection)	7 days	Regulates fatty acid metabolism	Reverses LPS-induced increase in fatty acids, regulates serum levels of linoleic acid	[223]
FGF19	Patient	1/3/6 mg (daily subcutaneous injection)	12 weeks, 24 weeks	Activation of the bile acid-FXR-FGF19 axis, activation of MAPK/ERK and PI3K/AKT signalling pathways	Improves hepatic lipid accumulation; boosts insulin sensitivity; regulates energy metabolism	[224]
FGF1	db/db & ApoE-KO mice	0.5 mg/kg (every-other-day intraperitoneal injection)	3 months	Inhibition of the activity and/or expression of lipogenic genes	Reduction in lipid deposition	[225]

note: MOD: model; Ref: references.

In the adipose tissue microenvironment, fibroblasts, as key components, not only participate in the dynamic remodelling of the ECM but also finely regulate the metabolism of adipocytes, the process of fibrosis, and the inflammatory response through the cAMP signalling pathway (including downstream effector molecules such as PKA/EPAC). In the cardiovascular system, the cAMP – EPAC pathway has a significant antifibrotic effect. The NO-cGMP signal produced by cardiac fibroblasts can regulate the cAMP level in cardiomyocytes across cells, and the activation of EPAC can effectively inhibit the proliferation of fibroblasts and the expression of α-smooth muscle actin induced by endothelin-1 [209,227]. The finding that beraprost. sodium alleviates myocardial fibrosis by upregulating the GSK-3β/p-CREB/cAMP pathway further confirms the crucial role of this pathway in maintaining the balance of myocardial lipid metabolism [210]. cAMP also determines the plasticity of fibroblast fate, and its analog 8-Br-cAMP can significantly increase the reprogramming efficiency of fibroblasts [211]. Under other pathological conditions, the adipose tissue microenvironment also undergoes significant changes. Burn eschar can stimulate the migration and proliferation of fibroblasts/adipose mesenchymal stromal cells while inhibiting angiogenesis [212]. Arecoline promotes oral submucous fibrosis through the PDE4A – cAMP/EPAC1 pathway, and this discovery provides a new target for the treatment of fibrotic diseases [213].

Collectively, these studies indicate that fibroblasts in adipose tissue – acting as core regulators of the microenvironment – synergistically interact with other metabolic pathways (e.g. FGFBP3 and AMPK) through the cAMP signalling network to dynamically regulate lipid synthesis, lipid breakdown, and energy metabolism. The underlying mechanisms involve paracrine signalling (e.g. FGFs), ECM remodelling, and immune – metabolic communication, suggesting potential therapeutic targets for obesity and related metabolic disorders.

### 6.3. Vascular endothelial (ve) cells

Adipose tissue is highly vascularized, with endothelial cells constituting the primary component of blood vessel walls. In the adipose microenvironment, endothelial cell signalling involves multiple key regulatory mechanisms. In recent years, the functional regulation of cAMP and its effectors (PKA and EPAC) in VE cells has received considerable attention. The cAMP – PKA/EPAC signalling pathway contributes to the formation and function of endothelial cells. As part of cAMP signal transduction, EPAC is becoming a research hotspot. A study demonstrated that EPAC1, but not EPAC2, is expressed in endothelial cells, indicating that EPAC1 plays an important role in regulating endothelial cell functions [214]. PGE2, which acts as a signal to activate the cAMP signalling pathway, elevates intracellular cAMP levels via PGE2 receptors. This, in turn, activates both the EPAC1/Rap1/Akt pathway and the PKA pathway. The EPAC and PKA signals converge at the level of GSK-3β phosphorylation, inhibiting the degradation of cytoplasmic β-catenin and promoting its nuclear accumulation. As a result, the expression of c-Myc and VEGF is upregulated, ultimately enhancing the proliferation of human umbilical cord blood-derived MSCs and promoting their differentiation into endothelial cells [215]. During blood vessel development, endothelial cAMP-dependent PKA regulates angiogenesis by controlling the number of tip cells, and PKA inhibition leads to excessive angiogenesis. One study indicates that in obesity, PKA inhibition leads to abnormal hyperplasia of the liver and adipose blood vessels [216]. This result indicates that endothelial PKA is not only a gatekeeper of endothelial cell activation during development but also in adult homoeostasis, thus preventing the abnormal reactivation of the angiogenesis program. In terms of the physiological functions of endothelial cells, cAMP mainly enhances the activity of endothelial nitric oxide synthase by activating PKA and EPAC, thereby increasing the release of nitric oxide and promoting vasodilation [214]. Additionally, PGE2 and its synthetic analogs can enhance the protective effect on the endothelial cell barrier and cytoskeleton remodelling through the PKA- and EPAC1/Rap1-dependent Rac activation pathways [217]. Similarly, studies have shown that the cAMP/EPAC/RAP1 axis is necessary for the efficient generation of endothelial cells with angiogenic potential [218]. CREB, as a downstream factor of the cAMP – PKA/EPAC signalling pathway, is activated by its regulated transcriptional coactivators – CRTC1 to CRTC3—which promote transcription by targeting the basic leucine zipper domain of CREB. CRTC2 is a major regulator of glucose metabolism in the liver and adipose tissue [219]. Under ischaemic conditions, CRTC2 plays a crucial protective role in endothelial vascular integrity [220]. Additionally, EPAC plays an important role in regulating inflammation and microvascular permeability caused by lipopolysaccharide [221]. In addition, PDE4D, by degrading cAMP, jointly regulates vascular permeability via the EPAC1/VE – cadherin complex [222]. Endothelial cells play indispensable roles in angiogenesis, homoeostasis, and immune responses under normal physiological conditions, and their dysfunction is closely related to pathologies such as cardiovascular diseases. Abnormal endothelial cell metabolism contributes to the development of many diseases [223]. In the field of pathological intervention research, the activation of the EPAC/Rap1 signalling pathway has shown unique value. A study has shown that the activation of the EPAC/Rap1 signalling pathway has a neuroprotective effect on brain damage caused by cerebral ischaemia – reperfusion through the restoration of the blood – brain barrier, which provides a new perspective for the treatment of cardiovascular diseases [224]. These findings suggest that cAMP plays a key role in angiogenesis, vasodilation, vascular permeability, and endothelial cell barrier protection through multiple pathways, such as the activation of PKA and EPAC.

Endothelial cells play dual roles in the adipose microenvironment. First, they act as effective barriers, particularly separating the blood from other components in adipose tissue. Second, VE cells can precisely regulate the transmembrane transport of bioactive molecules, metabolites, and other components through selective permeability mechanisms, thus maintaining the homoeostasis and biological functions of the adipose tissue microenvironment. The adipose microenvironment, which is constructed with the participation of endothelial cells, plays a crucial role in maintaining lipid homoeostasis by regulating the adipocyte differentiation process, influencing lipid metabolism, and modulating adipose inflammatory responses. Adipose endothelial cells play a crucial role in maintaining adipocyte metabolic homoeostasis by regulating insulin sensitivity, lipid turnover, and plasticity [225]. Endothelial cells also activate the brown fat differentiation program of progenitor cells (such as pericytes or MSCs) by secreting paracrine factors (such as insulin-like growth factor-binding protein 3 and IL-33) [228]. Brown adipocytes inhibit the formation of atherosclerotic plaques by increasing thermogenesis and lipid oxidation and reducing lipid deposition in blood vessel walls. VE cells produce CD73-dependent extracellular adenosine to regulate de novo lipogenesis in adipose tissue [229]. These findings indicate that endothelial cells and adipocytes complement each other in physiological processes and jointly maintain the body’s metabolic health. Under pathological conditions, endothelial cells act as amplifiers of tumour signals in fat and promote adipose tissue remodelling [230]. Additionally, CD146, a novel adipose receptor for angiopoietin-like protein 2 (ANGPTL2), binds to ANGPTL2 to activate CREB. While regulating adipogenesis, lipid metabolism, and energy consumption, it mediates the effect of ANGPTL2 on endothelial cells, thereby promoting adipose inflammation. These findings support the existence of reciprocal interactions between adipocytes and endothelial cells in the progression of adipose inflammation and obesity [231]. Therefore, endothelial cells play multiple crucial roles in their interaction with adipocytes. In turn, adipocytes, another key player in this bidirectional relationship, also exert a significant influence on endothelial cells. First, subcutaneous adipose-derived MSCs are more likely to differentiate into endothelial cells than visceral adipose-derived MSCs are, which may be related to differences in miRNA regulation [232]. Hypoxic conditions (such as those in obese adipose tissue) promote the endothelial differentiation of adipose-derived MSCs, but their function is immature [232]. These results indicate that the differentiation of endothelial cells is affected by multiple factors, including the condition of the adipose tissue (source and status). Additionally, deficiency of caveolin-1 (a lipid droplet outer shell protein) leads to an increase in endothelial cell PGI2 autocrine activity, followed by activation of the cAMP – PKA signalling pathway, promoting enhanced lipolysis [233]. These findings indicate that the structural characteristics of lipid droplets influence endothelial cell function, which in turn affects lipid metabolism. Moreover, different adipokines exert distinct effects on endothelial cell health. Some adipokines, including leptin, resistin, chemerin, and FABP4, exacerbate inflammation and arteriosclerosis, whereas adiponectin, FGF21, C1q/TNF-related protein 9, progranulin, omentin, and vaspin have cardioprotective effects, improving cardiovascular health by inhibiting inflammation and endothelial dysfunction [234,235]. Therefore, targeting specific adipokines is expected to provide hope for clinical intervention in diseases related to abnormal endothelial cell function, such as cardiovascular diseases. After binding to T-cadherin, adiponectin usually triggers a series of signal transduction events to promote the normal functions of endothelial cells, including the secretion of extracellular vesicles. Extracellular vesicles are crucial for maintaining the normal metabolic functions of adipocytes by transporting fatty acids, other plasma components, and signalling molecules and promoting the metabolism of healthy WAT. In obesity, the reduced binding of T-cadherin to adiponectin decreases the secretion of endothelial extracellular vesicles, hinders white fat metabolism, and thus exacerbates obesity [235]. Additionally, adiponectin targets the vascular endothelium and triggers organ-protective functions by binding to T-cadherin [236]. A clinical trial revealed that the percentage of endothelial cells in the stromal vascular fraction of adipose tissue in participants with a body mass index of 25 kg/m^2^ or higher is relatively low [237]. In addition, endothelial cells stimulate lipolysis in adipose tissue to support capillary growth, and this interaction pattern helps maintain normal metabolism in obese individuals [237]. These results indicate that the number or function of endothelial cells has a negative regulatory effect on overweight/obesity. However, the proinflammatory state of hypertrophic adipocytes in obesity is reflected in the activation of endothelial cells, thus promoting chronic inflammation [238]. The controversy surrounding the role of endothelial cells in obesity – whether they promote or inhibit disease progression – may arise from their dual regulatory functions in distinct physiological processes. These seemingly contradictory effects likely depend on the functional bias of endothelial cells within a specific microenvironment (e.g. metabolic vs. immune regulation), as well as dynamic shifts across different stages of obesity (early compensation vs. late decompensation). One study also demonstrated that adipose tissue from patients with lymphoedema exhibits endothelial cell dysfunction, resulting in increased vascular permeability and aberrant adipose hyperplasia, suggesting that such dysfunction may represent a core mechanism underlying abnormal adipose tissue remodelling [239]. Additionally, research in recent years has revealed the complex regulatory mechanisms of the chemokine network and vascular – immune interactions in the adipose tissue microenvironment. During adipose transplantation, C-X-C motif chemokine ligand (CXCL)13 plays a dual role. In the short term, its expression promotes angiogenesis by recruiting M2 macrophages and endothelial progenitor cells, which is beneficial for graft survival. However, sustained high expression over the long term may induce chronic inflammation, thereby reducing the long-term retention rate of grafts [240]. In the obese state, stromal cell-derived factor 1 (CXCL12) inhibits platelet-derived growth factor-B-mediated vascular remodelling through the C-X-C motif chemokine receptor4/7 pathway. This regulatory imbalance may lead to abnormal vascular function in adipose tissue, thereby affecting the normal expansion of adipose tissue [241]. The plant active ingredient Antcin K has significant cardiovascular protective effects. It reduces lipid deposition in macrophages, inhibits foam cell formation by upregulating Krüppel-like factor 4 expression, and simultaneously reduces the secretion of proinflammatory factors such as TNF-α and IL-1β, thus effectively improving endothelial function and alleviating the inflammatory response [242]. These findings not only clarify the key roles of chemokines such as CXCL13 and stromal cell-derived factor 1 in angiogenesis and inflammation regulation in adipose tissue but also provide an important theoretical basis for developing treatment strategies for metabolic diseases that target the chemokine network or the Krüppel-like factor 4 pathway, particularly new ideas for interventions related to obesity-associated vascular complications and postadipose-transplantation management.

Therefore, in the adipose tissue microenvironment, endothelial cells play a central role in maintaining lipid homoeostasis through multiple mechanisms, including regulating adipocyte differentiation, modulating lipid metabolism pathways, mediating inflammatory responses, and interacting with adipocytes in signalling. The key signalling pathway involved is cAMP – PKA/EPAC. These findings not only reveal the intrinsic links between endothelial cell dysfunction and the development of cardiovascular diseases, abnormal fat metabolism, and obesity but also provide innovative theoretical foundations and potential directions for early prevention, targeted treatment, and the development of new intervention strategies – offering promising breakthroughs in improving patients’ metabolic health.

**Figure 4.**
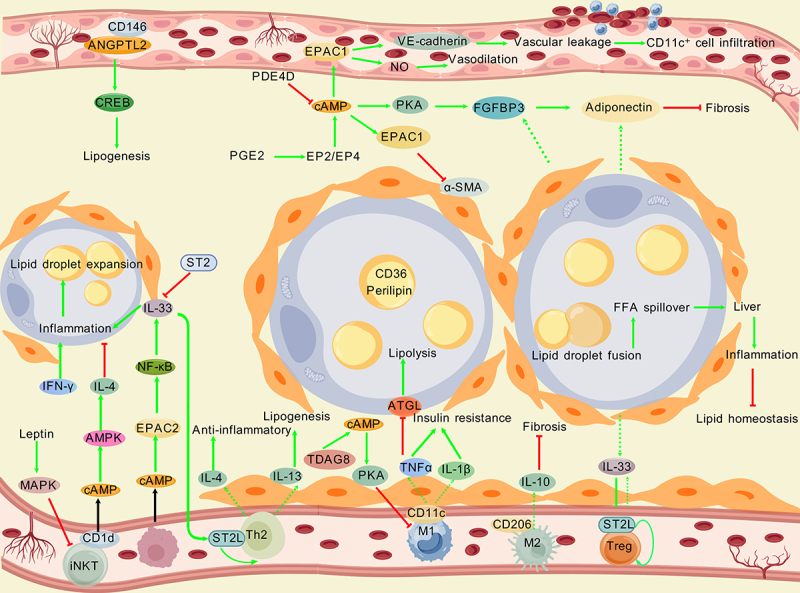
Regulatory network of the cAMP–PKA/EPAC signalling pathway in the adipose microenvironment created with BioGDP.com. In the adipose microenvironment, molecules like ANGPTL2 and CD146, alongside PDE4D (a regulator of cAMP production), initiate cAMP-dependent signalling: cAMP activates PKA and EPAC, which drive pathways (e.g. CREB, VEGFBP3) to control lipogenesis, lipolysis, inflammation (mediated by IL-33, NF-κB), and fibrosis. Immune cells – including iNKT cells and M1/M2 macrophages – further coordinate processes like insulin resistance, lipid droplet dynamics (expansion and fusion), and hepatic lipid homoeostasis. Together, this network ties cAMP signalling to the physiological and pathological regulation of the adipose microenvironment.

In summary, in the adipose microenvironment, immune cells, fibroblasts, and VE cells collaborate with each other to finely regulate lipid homoeostasis in the adipose microenvironment. The cAMP – PKA/EPAC signalling pathway is involved in the regulation of lipid homoeostasis in the adipose microenvironment (Figure 4). Once this regulation is imbalanced, it may trigger metabolic diseases such as obesity and diabetes.

Green solid lines: Pathway activation/promotion.

Green dashed lines: Secretion.

Black solid lines:Action execution.

→:Positive regulation/activation.

⊣ (red): Negative regulation/inhibition.

## 7. Targeting cAMP signalling for the treatment of metabolic diseases

### 7.1. PKA modulators

PKA, as a core effector of the cAMP signalling pathway, plays a complex and crucial role in the regulation of lipid metabolism. PKA regulates the lipolysis process in adipose tissue by phosphorylating various metabolism-related proteins, including hormone-sensitive lipase, perilipin, and adipose triglyceride lipase. In WAT, PKA activation promotes fat thermogenesis and lipolysis, releasing free fatty acids [243]. In the liver, PKA activation can inhibit fatty acid synthesis and promote gluconeogenesis [244]. Therefore, the bidirectional regulatory role of PKA in lipid homoeostasis may depend on tissue type and other factors. Selective PKA inhibitors, such as H89, KT5720, and Rp-8-Br – cAMPS, have demonstrated efficacy in improving abnormal lipid metabolism in basic research (Table 2 [245–262]). However, considering that the PKA signalling pathway has cardiovascular protective functions [263,264], the use of PKA inhibitors to treat diseases related to metabolic abnormalities may also face challenges such as tissue specificity and cardiovascular side effects. Recent research has demonstrated that specific subtypes of PKA, such as PKA – RIIβ, can regulate the WAT browning and participate in energy metabolism [117]. This breakthrough research not only expands our understanding of the functional specificity of PKA subtypes but also provides a theoretical basis for precise intervention in lipid metabolism regulation. We must consider the potential arrhythmogenic effects of RIIβ on cardiomyocytes, as well as the considerable interindividual variability in browning capacity. Future research should therefore prioritize the development of tissue-targeted PKA regulators. For example, adipose tissue – specific PKA inhibitors can reduce systemic lipolysis without impairing the function of other tissues. Similarly, subtype-selective activators (e.g. PKA – RIα- vs. RIIβ-specific compounds) could be explored. Additionally, the development of allosteric regulators that bind to the regulatory subunit (R subunit), rather than the catalytic subunit (C subunit), of PKA may represent a promising direction for future investigations.

**Table 2.** PKA modulators (inhibitors/antagonists and agonists) related to lipid homoeostasis.

Inhibitor/antagonist						
Inhibitor name	Disease type	MOD	Dose	Duration	Core effect	Ref.
H89	Not applicable	Yellow catfish Pelteobagrus fulvidraco	30 μM	18 h	Reduced the activity of lipolysis-related enzymes in lipid metabolism.	[245]
H-89	Obesity	3T3-L1 adipocytes	20 μM	1 h pretreatment +24 h incubation	Suppressed the lipolytic activity	[246]
H89	Not applicable	3T3-L1 adipocytes	Not applicable	Not applicable	Attenuated musin’s effect (alleviates lipid accumulation) on lipid metabolism	[247]
H89	Not applicable	Grass carp adipocytes	20 mM	3 h pretreatment +48 h incubation	weakened DHA-induced lipolysis	[248]
H-89	Obesity and obesity-related fatty liver degeneration	HepG2 cells	20 μM	1 h pretreatment +24 h incubation hours	Reversed UCP1/PGC-1α expression; increased lipid accumulation	[249]
H-89 dihydrochloride	Obesity-related hepatic steatosis	3T3-L1 adipocytes	30 μM	Not mentioned	Reduced PGC-1α/UCP1 (brown-labelled proteins) in DZF-treated cells	[250]
H89	Not applicable	Grass carp adipocytes	5 μM	48 hours	Alleviated ER stress-induced lipolysis	[251]
H89	Not applicable	3T3-L1 adipocytes	Not applicable	Not applicable	Eliminated *Humulus japonicus* extract’s effect on lipid metabolism	[252]
H89	Obesity	Porcine adipocytes	10 μM/L	24 h	Affected the phosphorylation of perilipin and hormone-sensitive lipase	[253]
H89	Obesity	Mouse brown adipocytes	10 µM	Not mentioned	Inhibited lipolysis	[254]
KT5720	Metabolic syndrome and obesity-associated complications	Male Wistar rats	1 μmol/kg (intraperitoneal)	Twice	Inhibited Na_2_S-induced NEFA & glycerol increase	[255]
KT5720	Diet-induced obesity	Mouse white adipocyte	1 μM	Not mentioned	Influencing genes like UCP1 and PGC1α	[256]
KT5720	Obese diabetic	3T3-L1 adipocytes	1 h prior to cilostazol treatment	Not mentioned	Exacerbated insulin resistance	[257]
KT5720	Not applicable	3T3-L1 adipocytes	80 µM	Not mentioned	Prevented isoproterenol-induced FSP27 elevation	[258]
Rp-8-Br −cAMPs	Obesity	Mice models and C3H10T1/2 adipocytes	100 μM	Not mentioned	Reversed enhanced lipolysis and reduced lipid accumulation	[259]
H89			5 μM			
Rp-8-Br-cAMPs	Diabetes mellitus	Rat β cells	100 mmol/l	Not mentioned	Impaired insulin secretion, exacerbated β-apoptosis, and indirectly affected lipid metabolism	[260]
Rp8CTP	Metabolic-dysfunction-associated liver disease	Primary rat hepatocytes	Not mentioned	Not mentioned	Partially eliminated caffeine’s protective effect against palmitic acid-induced hepatocyte lipotoxicity	[261]
Agonist						
Agonist name	Disease type	MOD	Dose	Dration	Core effect	Ref.
6-Bnz-cAMPs	Obesity	Obesity mice models and C3H10T1/2 adipocytes	100 µM	Not mentioned	Promoted HSL/perilipin phosphorylation, enhancing lipolysis and reducing fat accumulation	[259]
Sp-cAMP	Diabetes and obesity	Rats	30 μmol/L	Not mentioned	Reduced rat glucose production; enhanced vagal afferent nerve conduction	[262]

note: MOD: model; Ref: reference.

Additionally, given the bidirectional regulation of the cAMP – PKA pathway in lipid homoeostasis, only a handful of studies have investigated PKA agonists (Table 2). However, most existing research relies on cAMP analogs, which may lack specificity in their mechanism of action, thereby limiting their translational importance. Consequently, current and future investigations will focus primarily on the development and application of PKA inhibitors or antagonists.

### 7.2. EPAC inhibitors

In recent years, with the in-depth study of the cAMP signalling pathway, the role of EPACs (including EPAC1 and EPAC2) in metabolic regulation has gradually attracted attention. As an effector molecule of cAMP, EPAC plays a key role in adipogenesis, lipolysis, insulin sensitivity, and hepatic lipid metabolism. Therefore, the use of small-molecule inhibitors targeting EPAC has become a potential treatment strategy for regulating lipid metabolism disorders. Currently, various EPAC-selective inhibitors have been developed, including mainly small-molecule compounds such as those in the ESI series (ESI-09 and ESI-05) and CE3F4 (Table 3 [265–294]). These inhibitors competitively bind to the cAMP-binding domain of EPAC, blocking its conformational changes and the activation of downstream effector molecules (such as Rap1). EPAC subtypes exhibit significant tissue-specific characteristics related to metabolic regulation. A study has shown that EPAC1 is expressed mainly in adipose tissue, promoting the formation of beige/brown fat and energy consumption and counteracting diet-induced obesity [87,295,296]. Its knockout can lead to a reduction in brown fat and exacerbation of obesity [115]. Moreover, EPAC1 is involved in the development of atherosclerosis by regulating the uptake of oxidized low-density lipoproteins [157]. EPAC2 is highly expressed in mainly pancreatic β-cells and is involved in insulin secretion [297]. Systemic inhibition may interfere with glucose metabolism. Therefore, developing subtype-selective inhibitors is crucial for precisely regulating lipid metabolism. However, as EPAC shares the upstream cAMP signal with PKA, the subtype selectivity and off-target effects of inhibitors still need further optimization. Additionally, EPAC inhibitors may face many challenges in translational medicine research, including pharmacokinetic limitations. For example, existing inhibitors (such as ESI-09) have low oral bioavailability, and their efficacy may be affected by rapid metabolism. Moreover, owing to the widespread expression of EPAC1 and EPAC2 in various tissues, systemic inhibition by inhibitors with insufficient tissue targeting may lead to unexpected effects (such as affecting the functions of pancreatic islet β-cells). Future research on EPAC inhibitors could be based on rational drug design of the EPAC protein structure to improve selectivity and stability and develop tissue-specific delivery systems (such as liver-targeted nanoparticles). Additionally, given the complexity of the cAMP pathway, EPAC inhibitors may be combined with GLP-1 receptor agonists or PPARγ regulators to synergistically improve metabolic abnormalities. All of these findings require further basic and clinical research for further exploration.

**Table 3.** Known EPAC modulators.

EPAC Inhibitor/antagonist		
Inhibitor Names	Target Molecule	Ref.
(R)-CE3F4	EPAC1	[255]
ESI-09 3-(5-tert-butyl-isoxazol-3-yl)-2-[(3-chloro-phenyl)-hydrazono]-3-oxo-propionitrile (ESI-09)	EPAC1 and EPAC2	[256–260]
N,N-Diphenylamines	EPAC2	[261]
NY0123	EPAC1	[262]
ESI-05	EPAC2	[263,264]
AM-001 (a thieno [2,3-b]pyridine derivative)	EPAC1	[265,266]
A thiobarbituric acid derivative, 5,376,753	EPAC	[267]
CE3F4 (A tetrahydroquinoline analog)	EPAC1	[268]
HES-1–09 ZL0524	EPAC1and EPAC2	[269]
AM-001	EPAC1	[270]
MAY0132, RDR02145, AAK-399 and AAD-026	EPAC2	[271]
12a	EPAC	[272]
ESI-05	EPAC2	[273]
brefeldin A	EPAC	[274]
ESI-07	EPAC2	[275]
Brefeldin A variant	EPAC2	[276]
Agonist		
Agonist name	**Target Molecule**	Ref.
I942	EPAC1	[277–279]
8CPT-2Me-cAMP	EPAC	[280]
8-pCPT-2’-O-Me-cAMP	EPAC	[281]
Sp-8-BnT-cAMPS	EPAC2	[282]
Φ-O-Me-cAMP	EPAC1	[283]
8-(4-chlorophenylthio)-2’-O-methyladenosine-3’,5’−cyclic monophosphate	EPAC	[284]

note: Ref: references.

### 7.3. PDE inhibitors

The PDE superfamily consists of 11 subtypes (PDE1–11), which finely regulate lipid metabolism by hydrolysing cAMP and cGMP. Many studies have reported the various pharmacological effects of PDE inhibitors in the treatment of metabolic diseases.

Vinblastine is a PDE1 inhibitor that can increase the levels of cAMP and cGMP. On the one hand, it prevents adipocyte differentiation by inhibiting cell signalling related to early adipocyte differentiation. On the other hand, it increases lipolysis and the expression of UCP1 by increasing the cAMP level, thus inhibiting lipid accumulation [298]. Cilostazol is a selective PDE3 inhibitor that can increase intracellular cAMP, promote the browning of visceral WAT, and inhibit gluconeogenesis [299,300]. The non-specific PDE inhibitor 3-isobutyl-1-methylxanthine can also inhibit gluconeogenesis through the cAMP pathway [300]. In terms of obesity metabolic regulation, research has shown that PAN – PDE4 inhibitors such as roflumilast can reduce high-fat diet-induced obesity and improve glucose metabolism and insulin sensitivity, independent of reduced food intake or increased physical activity [301]. It has better short-term weight loss effects than metformin in obese women with polycystic ovary syndrome, which has certain clinical translational value [301]. Genetic study has shown that the knockout of PDE4B and PDE4D subtypes can replicate these metabolic improvement effects, whereas the knockout of PDE4A/C has no such effect [301]. Similarly, the PDE4 inhibitor rolipram exerts weight loss effects by reducing food intake, increasing energy consumption, and improving leptin sensitivity [26]. With the development of PDE inhibitors, some new inhibitors have also become new favourites for researchers. The PDE4B inhibitor A-33 and its derivative MDL3 (which inhibits both PDE4B and PDE5A) significantly improve chronic liver disease and adipose tissue remodelling, and MDL3 can also restore glucose sensitivity in obese mice [302]. Treatment of high-fat diet-induced obesity in mice with the PAN – PDE4 inhibitor roflumilast can be achieved by reducing white fat pads [25]. These results suggest that inactivation of PDE4 represents a promising approach to address obesity and related metabolic abnormalities, such as elevated blood glucose levels. In BAT, the combined use of PDE3 and PDE4 inhibitors has a synergistic effect on activating thermogenic functions. Under basal conditions, simultaneous inhibition of PDE3 and PDE4 is required to induce UCP1 mRNA expression and lipolysis. Under β-adrenergic stimulation, a PDE3 inhibitor alone can increase UCP1 expression, and a PDE4 inhibitor specifically promotes lipolysis [303]. PDE5 inhibitors have thermogenic, weight-reducing, and insulin-sensitizing effects on high-fat diet-induced obesity [304]. However, in adipocytes and hepatocytes, the increase in cGMP mediated by the PD5 inhibitor sildenafil inhibits PDE3 at lower concentrations, thus increasing cAMP. At higher concentrations, sildenafil further increases cGMP levels, which in turn activates PDE2 and reduces cAMP. These findings indicate that in adipocytes and hepatocytes, crosstalk occurs between cAMP and cGMP through PDE2, PDE3, and PDE5, and the coordinated regulation of PDE2/3/5 has been confirmed to be involved in energy sensing [305]. These findings not only reveal the spatiotemporal-specific roles of different PDE subtypes in metabolic tissues but also provide a theoretical basis for the development of tissue-targeted drugs or subtype-selective inhibitors. Other PDE subtypes also show potential for metabolic regulation. In clinical trials, treatment with the novel PDE10A inhibitor MK-8189 significantly reduced body weight, particularly in patients with obesity [306]. THPP-6, a small-molecule PDE10A inhibitor used to treat mice on a high-fat diet, leads to reduced food intake, weight loss, and decreased obesity [307]. However, the side effects of existing PDE inhibitors – such as nausea and vomiting caused by PDE4 inhibitors and possible ocular and auditory adverse reactions from PDE5 inhibitors – suggest that more precise targeting strategies are needed [308–310]. Future research should focus on the development of allosteric regulators, the optimization of subtype-specific inhibitors, and the development of precision drug delivery systems on the basis of tissue distribution characteristics to achieve a balance between the safety and effectiveness of metabolic disease treatment.

### 7.4. cAMP modulators

The overall regulatory strategies of the cAMP signalling pathway involve multilevel intervention measures. At the receptor level, targeting GPCRs such as the β3-adrenergic receptor (mirabegron) and GLP-1 receptor (liraglutide) can regulate cAMP production [311,312]. At the molecular level, cAMP analogs (such as 8-Br-cAMP, Sp-8-Br-cAMPS, Sp-5,6-DClcBIMPS, 8-AHA-cAMP, and 8-CPT-cAMP) and allosteric modulators can directly affect downstream effectors [313–315]. In recent years, new cAMP regulatory strategies have emerged. Bicyclic nucleotide analogs can selectively activate the PKA or EPAC pathway [316]. Photosensitive cAMP prodrugs achieve spatiotemporal-specific regulation [317]. Nanocarrier-delivered cAMP mimetics improve tissue targeting [318]. The development of allosteric modulators is particularly noteworthy. Although a few allosteric modulators of cAMP have been identified – such as propofol, an anaesthetic agent that inhibits HCN channels by allosterically modulating the cAMP-dependent gating mechanism – there are limited reports in the lipid-related field. Therefore, developing cAMP allosteric agents for lipid homoeostasis disorders may offer new opportunities for the prevention and treatment of related diseases. Additionally, PKA subtype-selective regulators can enable precise modulation of specific signalling branches [319]. However, only a few studies have explored this approach in the context of lipid metabolic diseases, suggesting a promising direction for future research. Additionally, the CRISPR/dCas9 system enables the precise targeting of specific gene loci. The recruitment of epigenetic modification enzymes can induce the methylation or acetylation of key genes in the cAMP signalling pathway (e.g. AC and PKA catalytic subunit genes), thereby achieving long-term regulation of gene expression at the transcriptional level. A study has used this technology to demonstrate that the β-catenin – encoding gene *CTNNB1* promotes obesity by modulating the relationship between preadipocytes and mature adipocytes [320], potentially offering new avenues for the prevention and treatment of obesity and related diseases. Furthermore, combining CRISPR/dCas9 with gene therapy allows in vivo delivery via vectors to stably modulate the cAMP signalling pathway, providing a potential long-acting strategy for treating disorders of lipid homoeostasis – such as obesity – caused by dysregulated cAMP signalling.

### 7.5. Ac modulators

The adenylyl cyclase family plays an important role in the regulation of lipid homoeostasis. Research has shown that SQ22536 (SQ), an adenylyl cyclase inhibitor, can weaken the effect of acacetin-promoted white fat browning by inhibiting the AC – cAMP – PKA signalling pathway [63]. Therefore, targeted regulation of AC may be an important approach for treating obesity and related diseases. However, in in vivo and in vitro experiments, different subtypes of AC regulate metabolic processes through unique mechanisms. A study found that mice lacking Adcy3 (the gene encoding AC3) show increased visceral fat accumulation when fed a normal diet or a high-fat diet and develop abnormal metabolic characteristics, such as reduced insulin sensitivity, dyslipidemia, and an increase in proinflammatory cytokines [321]. Additionally, cysteine deficiency in Adcy3 leads to decreased expression of genes related to thermogenesis, fatty acid oxidation, and the insulin signalling pathway while promoting the expression of genes related to adipogenesis [321]. These results suggest that in peripheral tissues, the cAMP signal generated by Adcy3 May play an important role in regulating obesity and insulin sensitivity. Moreover, in BAT, AC3 negatively regulates cAMP synthesis through the cold-induced truncated isoform AC3–AT, limiting excessive thermogenesis and maintaining energy balance [322]. Therefore, Adcy3 (AC3) may be a potential target for preventing or treating obesity and related metabolic complications. Additionally, AC5-knockout mice are resistant to glucose intolerance, insulin resistance, and obesity when exposed to a high-fat diet. Moreover, they improve energy metabolism efficiency, particularly in skeletal muscle, where mitochondrial function is enhanced and oxidative stress is reduced. This may be the mechanism for improving glucose tolerance and insulin sensitivity. Thus, AC5 is proposed as a potential new target for the treatment of obesity and diabetes [323]. However, other complementary studies suggest that Adcy5-deficient mice exhibit an improved metabolic state under certain conditions, such as smaller adipocytes and a different adipose tissue gene expression pattern, which may be related to better insulin sensitivity. Nevertheless, in many cases, these mice do not exhibit significant physiological or biochemical benefits. For example, under a high-fat diet, female mice lacking Adcy5 have enlarged adipocytes, indicating that the interaction between diet and genotype may affect cholesterol metabolism [324]. Overall, Adcy5 deficiency does not provide the expected protective effects in all aspects, particularly in terms of improving glucose tolerance and insulin sensitivity under certain dietary and sex conditions. These results suggest that targeting Adcy5 does not necessarily improve insulin sensitivity, which may limit its relevance as a potential drug target. Furthermore, the activation of AC6 can alter the endocytic pathway of TLR4, accelerate its degradation, and thus inhibit TLR4 signalling, playing a negative regulatory role in TLR4 signalling [325]. These findings may provide a new treatment method for limiting inflammatory responses, particularly for obesity-related inflammatory diseases. The localization and function of AC8 are regulated by the palmitoylation of AKAP79 (PKA-anchoring protein 79) in lipid rafts, whereas soluble AC10 (sAC/ADCY10) directly promotes thermogenesis in BAT [326,327]. These findings reveal that AC subtypes play multilevel roles in lipid metabolism, energy balance, and inflammation by spatiotemporally regulating cAMP signals, providing potential targets for the treatment of metabolic diseases. Therefore, developing regulators that target different AC subtypes may be helpful in improving insulin resistance, promoting fat browning, and alleviating metabolic inflammation. However, their clinical application still faces challenges such as subtype functional compensation, insufficient tissue targeting, and individual differences. Future research should focus on developing highly selective inhibitors on the basis of the crystal structures of AC subtypes, exploring the synergistic or antagonistic effects of different AC subtype regulators on lipid homoeostasis, constructing tissue-specific drug delivery systems, and deeply exploring combination treatment plans with other metabolic regulators to fully exploit the potential of these drugs in the treatment of metabolic diseases.

## 8. Discussion/conclusion

In summary, the cAMP – PKA/EPAC signalling pathway, as a core regulatory network of cellular energy metabolism, plays an irreplaceable role in maintaining lipid homoeostasis. This pathway plays a dual role in lipid regulation, with outcomes potentially depending on the target tissue type, metabolic state, and activation status of the signalling pathway. Numerous studies have shown that dysregulation of the cAMP – PKA/EPAC pathway is closely associated with the onset and progression of various metabolic diseases – including obesity and diabetes – highlighting its potential as a therapeutic target. Despite notable advances, several critical challenges remain. First, the mechanism underlying the interaction between PKA and EPAC signals during dynamic metabolic transitions – particularly in the feeding – fasting cycle – is still poorly understood, and systematic studies on their coordinated regulation are lacking. Second, most current studies focus on individual tissues or cell types, limiting our knowledge of interorgan crosstalk mediated by this pathway. Specifically, the cAMP-dependent communication network between adipose tissue and the liver remains to be elucidated. To elaborate on this point, the adipose-liver axis represents a critical endocrine circuit in systemic lipid homoeostasis, and the cAMP pathway is poised to be a key mediator. During fasting, catecholamine-stimulated cAMP signalling in adipose tissue promotes lipolysis, releasing FFAs and glycerol into the circulation. The liver then utilizes these substrates for gluconeogenesis and ketogenesis. However, the role of cAMP in the liver during this process is complex and context-dependent. It remains unclear how cAMP signals in hepatocytes, potentially modulated by adipose-derived factors, integrate with incoming lipid fluxes to orchestrate an appropriate metabolic response. Conversely, in overnutrition and obesity, aberrant cAMP signalling in both tissues may contribute to metabolic dysfunction: impaired cAMP-mediated lipolysis in adipose tissue can lead to ectopic lipid accumulation in the liver, while dysregulated hepatic cAMP/PKA signalling may exacerbate gluconeogenesis and steatosis. Key unanswered questions include: (1) What are the specific cAMP-dependent hepatokines or adipokines that facilitate this cross-talk? (2) How does the cAMP-EPAC/PKA balance in one tissue influence the signaling status and metabolic function of the other? Elucidating these mechanisms will be crucial for understanding the pathophysiology of diseases like NAFLD and for developing targeted therapies. Third, at the technical level, there is a lack of high-spatiotemporal-resolution imaging techniques capable of monitoring cAMP – PKA/EPAC signaling dynamics in vivo in real time, greatly hindering our ability to characterize its regulatory features. Moreover, although some pharmacological agents targeting this pathway (e.g. PDE inhibitors) have progressed to preclinical evaluation, their tissue specificity and long-term safety require further validation. Specifically, preclinical advances in cAMP – PKA/EPAC pathway-targeted therapies for metabolic diseases have not yet translated smoothly to clinical applications due to three key barriers. First, species-specific differences in pathway regulation exist between animal models and humans – for instance, the expression pattern of EPAC isoforms in adipose tissue varies between mice and humans, which may lead to inconsistent therapeutic effects in clinical trials [328,329]. Second, off-target effects of current candidate drugs (e.g. non-selective PDE inhibitors) limit their clinical application, as they may disrupt cAMP signalling in non-target tissues (e.g. cardiac tissue) and cause adverse reactions [330]. Additionally, the complexity of human metabolic disorders (e.g. comorbidities such as diabetes and hypertension) cannot be fully recapitulated by animal models, further hindering the translation of preclinical findings. Notably, much of the current research remains at the in vitro or animal model stage, and these models cannot fully recapitulate the human microenvironment, translating findings into clinical applications remains difficult. Therefore, high-quality, large-scale clinical cohort studies are urgently needed to bridge the gap between preclinical research and clinical application, while addressing the aforementioned translational challenges.

Given the increasing incidence of metabolic diseases, several promising research directions deserve in-depth investigation. At the basic research level, emerging technologies such as single-cell sequencing, spatial transcriptomics, and epigenetics are expected to clarify the precise regulatory mechanisms of the cAMP – PKA/EPAC pathway in specific cellular subsets. Technically, the development of genetically encoded fluorescent reporters to monitor real-time cAMP dynamics in target tissues will significantly advance our understanding of its temporal and spatial regulatory processes. In translational medicine, the design of tissue-specific modulators of the cAMP pathway, combined with exploration of their synergy with other metabolic networks, may offer breakthroughs in disease treatment. Furthermore, elucidating how environmental factors – such as diet and exercise – modulate this pathway via epigenetic mechanisms may reveal new molecular targets for lifestyle-based interventions. Finally, large-cohort clinical studies are essential to bridge the gap between basic research and clinical application. These efforts will not only deepen our understanding of lipid metabolism but also pave the way for precision therapies for metabolic disorders.
